# Tendon Cells Root Into (Instead of Attach to) Humeral Bone Head via Fibrocartilage-Enthesis

**DOI:** 10.7150/ijbs.79007

**Published:** 2023-01-01

**Authors:** Zheng Wang, Chi Ma, Diane Chen, Caitlin Haslett, Chunmei Xu, Changchun Dong, Xiaofang Wang, Minghao Zheng, Yan Jing, Jian Q. Feng

**Affiliations:** 1 Department of Biomedical Sciences, Texas A&M University College of Dentistry, Dallas, Texas 75246, USA.; 2 Department of Orthopaedic Surgery, University of Texas Southwestern Medical Center, Dallas, TX, 75219, USA.; 3 Centre for Orthopaedic Research, Medical School, The University of Western Australia, Nedlands, WA, Australia.; 4 Department of Orthodontics, Texas A&M University College of Dentistry, Dallas, TX, 75246, USA.; 5 Dental School and Oral Health Centre, The University of Western Australia, Nedlands, 6009 Australia.

**Keywords:** Tendon cell, Scx, Cell lineage tracing, Fibrocartilaginous enthesis

## Abstract

Large joints are composed of two closely linked cartilages: articular cartilage (AC; rich in type II collagen, a well-studied tissue) and fibrocartilaginous enthesis (FE; rich in type I collagen, common disorder sites of enthesopathy and sporting injuries, although receiving little attention). For many years, both cartilages were thought to be formed by chondrocytes, whereas tendon, which attaches to the humeral bone head, is primarily considered as a completely different connective tissue. In this study, we raised an unconventional hypothesis: tendon cells directly form FE via cell transdifferentiation**.** To test this hypothesis, we first qualitatively and quantitatively demonstrated distinct differences between AC and FE in cell morphology and cell distribution, mineralization status, extracellular matrix (ECM) contents, and critical ECM protein expression profiles using comprehensive approaches. Next, we traced the cell fate of tendon cells using Scx^Lin^* (*a tendon specific* Cre Scx^CreERT2^; R26R-tdTomato* line) with one-time tamoxifen induction at early (P3) or young adult (P28) stages and harvested mice at different development ages, respectively. Our early tracing data revealed different growth events in tendon and FE: an initial increase but gradual decrease in the Scx^Lin^ tendon cells and a continuous expansion in the Scx^Lin^ FE cells. The young adult tracing data demonstrated continuous recruitment of Scx^Lin^ cells into FE expansion during P28 and P56. A separate tracing line, 3.2 Col 1^Lin^ *(*a so-called "bone-specific" line), further confirmed the direct contribution of tendon cells for FE cell formation, which occurred in days but FE ECM maturation (including high levels of SOST, a potent Wnt signaling inhibitor) took weeks. Finally, loss of function data using diphtheria toxin fragment A (DTA) in Scx^Lin^ cells demonstrated a significant reduction of Scx^Lin^ cells in both tendons and FE cells, whereas the gain of function study (by stabilizing β-catenin in Scx^Lin^ tendon cells via one-time injection of tamoxifen at P3 and harvesting at P60) displayed great expansion of both Scx^Lin^ tendon and FE mass. Together, our studies demonstrated that fibrocartilage is an invaded enthesis likely originating from the tendon via a quick cell transdifferentiation mechanism with a lengthy ECM maturation process. The postnatally formed fibrocartilage roots into existing cartilage and firmly connects tendon and bone instead of acting as a simple attachment site as widely believed. We believe that this study will stimulate more intense exploring in this understudied area, especially for patients with enthesopathy and sporting injuries.

## Introduction

An enthesis (also called an osteotendinous or osteoligamentous junction) is the attachment of a tendon, ligament, or joint capsule to bone 1. This specialized “organ” is a solid and stable anchor for musculoskeletal movement and functions by resisting the mechanical loading transferred from soft to hard tissue 2, 3. There are two types of entheses: fibrous and fibrocartilaginous. Their distribution depends on the character of the tissue at different bone interfaces 4, 5. Fibrous entheses are located on the middle shafts of long bones, which are thought to be formed by fibroblasts. Fibrocartilaginous entheses attach to the end of bones, such as at the epiphyses, which are made of fibrocartilage cells.

Histologically, fibrocartilaginous entheses are composed of 4 zones: dense fibrous connective tissue, uncalcified fibrocartilage, calcified fibrocartilage, and bone 6. The calcified- and non-calcified fibrocartilage zones are divided by a line called the tidemark. It is widely believed that the enthesis fibrocartilage cells originate from a population of mesenchymal progenitor cells, as these enthesis cells co-express Scx (a transcriptional factor highly expressed tendon cells) and Sox9 (a transcriptional factor of chondrogenesis) 7-11. In addition, these cells express Gli1 (an early mesenchymal transcription factor) and respond to hedgehog signaling during growth and injury healing processes 12-14. Interestingly, enthesis cells express high levels of collagen type II and proteoglycans such as aggrecan 15 as well as growth and differentiation factor 5 (Gdf5) 16. Furthermore, the phenomenon of existing cartilage being eroded on the bone side and replaced with fibrocartilage on the tendon side during growth is akin to the behavior of a “growth plate”; this idea was proposed by Benjamin and Ralphs 15 and echoed by Thomopoulos 2. However, there is largely a lack of experimental evidence behind this theory.

Cell transdifferentiation from mesenchymal cells into epithelial cells (or vice versa) occurs in numerous developmental events such as gastrulation, neural crest, and somite dissociation, craniofacial development, wound healing, organ fibrosis, and tumor metastasis 17. Recent publications from us and others showed that the direct cell transdifferentiation from one mature cell such as a hypertrophic chondrocyte to another type of terminated cell like an osteoblast/ osteocyte plays a critical role during postnatal skeletal formation without undergoing the stem cell stage 18-27. Similarly, fish tendon forms a fibrocartilaginous pad 27, and mouse tendon cells directly transdifferentiate into a subset of chondrocytes in TMJ 28. Moreover, tendon calcifies in birds under tensile loading 29, 30. *In vitro* and *ex vivo* data also showed multiple cell fates of tendon progenitor cells, including forming cartilage tissue 31. In humans, calcific tendonitis occurs either through degenerative or reactive calcification 32-35. However, it is largely unclear if tendon lineage cells directly contribute to fibrocartilage enthesis formation.

Clinically, the fibrocartilaginous enthesis is vulnerable to overuse injuries, especially in large joints such as the shoulder joint 3, 4. Given its poor regenerative ability, damage to the enthesis fibrocartilage usually causes permanent tissue loss and disability. Interestingly, the presence of fibrocartilage within tendons was reported as synonymous with mucoid degeneration and considered a prelude to tendon rupture or calcifying tendinitis 36. Degeneration and tearing of tendons are associated with pronounced fibrocartilaginous metaplasia, perhaps due to enhanced activity of matrix metalloproteinases 37, 38. Adult tendon cells were found to undergo aberrant differentiation into cartilage and bone cells after injury 35. Current clinical treatment approaches for fibrocartilage injuries are very limited; conservative or surgical interventions usually fail and/or cause further tissue damage 39-41.

In this study, we attempted to address whether the enthesis-fibrocartilage entity is an isolated niche (similar to a growth plate) or originates from adjacent cells such as tendon cells using the humerus as a model with multiple comprehensive techniques, including different cell lineage tracing lines. Our results for the first time demonstrated that the enthesis fibrocartilage, containing a unique mixture of tendon-cartilage-bone ECMs with a high level of SOST (a potent inhibitor of Wnt-β-catenin signaling, originate from Scx+ tendon cells via a cell transdifferentiation mechanism. Ablation of the Scx^Lin^ tendon cell population drastically reduced the enthesis fibrocartilage mass using the Scx^CreERT2^; R26R-DTA (diphtheria toxin A) line. Thus, we propose that the enthesis fibrocartilage originates from Scx^Lin^ tendon cells, which “invade” and grow into the existing cartilage and bone of humerus epiphyses, making the tendon-bone interface an integrated continuum rather than a simple attachment.

## Results

### Distinct differences between enthesis fibrocartilage (the least studied tissue) and articular cartilage (a well-studied tissue partly due to a link to arthritis) at light microscopy levels

**Figure 1:**
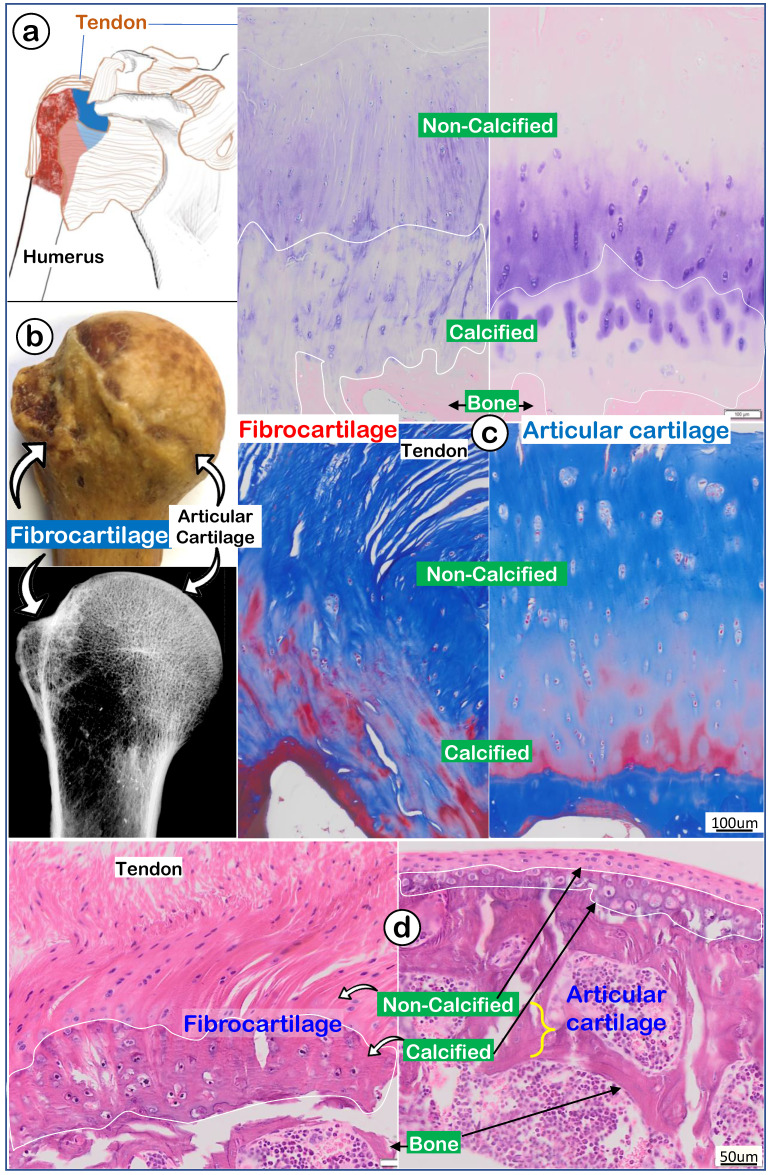
** A very thin human subchondral bone layer underneath of the thick fibrocartilage versus a thick subchondral bone layer under the thin articular cartilage layer. (a)** Humerus head is surrounded by numerus tendon; **(b)** a 97-year-old humerus head photograph and x-ray; and **(c)** images of toluidine blue stain (upper panels) and Masson's trichrome stain (lower panels) to show differences between fibrocartilage (left panels) and articular cartilage (right panels); and **(d)** a non-decalcified H&E stain image from an adult mouse humerus head.

To better address the largely unknown features of enthesis fibrocartilage, we started from the human shoulder joint with a drawing that depicted the humerus head (containing both fibrocartilage and articular cartilage) wrapped by numerous muscle tendons (Figure 1a). A photograph image (Figure 1b, *upper panel*) and a radiograph image (Figure 1b, *lower panel*) of a 97-year-old humerus head displayed a rough fibrocartilage surface and smooth articular cartilage. Toluidine blue staining images (Figure 1c, *left panel*) and Masson's Trichrome staining images (Figure 1c, *left panel*) showed a fiber-like structure in the fibrocartilage zone compared to a smooth structure in articular cartilage (Figure 1c, *right panel*). H&E staining images revealed a large non-calcified area (full of column-like cells with rich areas of fibers) plus a calcified area (with much larger cell bodies but few fibers; Figure 1d, *left panel*). In contrast, there was a thin strip of articular cartilage mass (Figure 1d, *right panel*).

**Figure 2:**
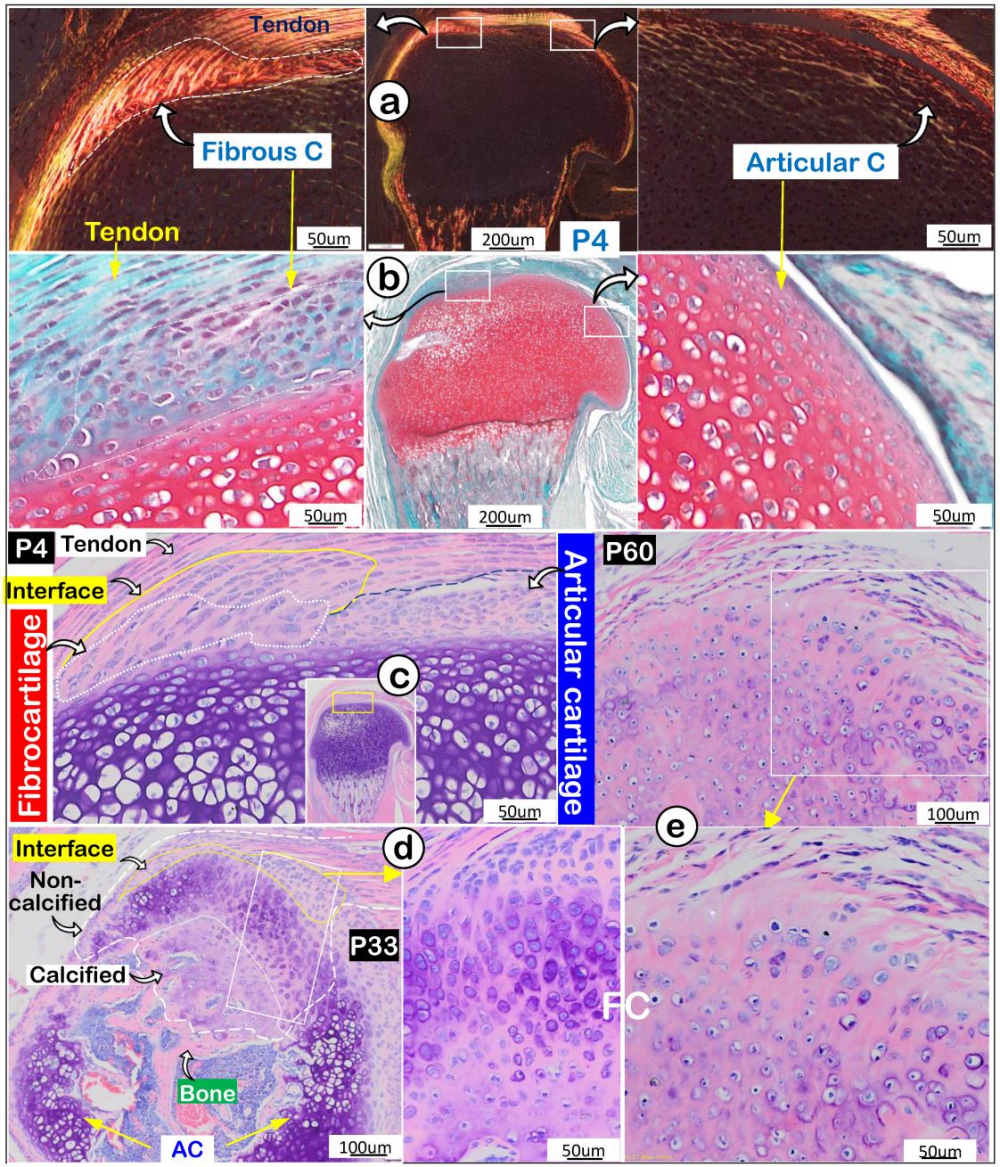
** Invasion and expansion of fibrocartilage (FC) compared to articular cartilage (AC) during mouse humerus postnatal development. (a)** Polarized light images showed strong collagen fibers in the fibrocartilage (*left panel*); **(b)** Safranin O stain images reveled the newly formed FC cells compared to AC cells (right panel); **(c)** Toluidine Blue stain images revealed the newly formed interface and fibrocartilage on the existing cartilage at postnatal day 4 (P4); **(d)** Toluidine Blue stain images at P33 revealed the non-calcified and calcified fibrocartilage; and (e) Toluidine Blue stain images showed a massive expansion of fibrocartilage at age of P60.

Next, we compared features of enthesis fibrocartilage and articular cartilage during mouse development starting at postnatal day 4 (P4). Our polarized light images showed a different type I collagen fiber distribution pattern from that in tendon with few type I collagen fibers in articular cartilage (Figure 2a). Safranin O staining images (richness in glycogen and glycoproteins) revealed a negative stain in newly formed fibrocartilage (Figure 2b;* negative panel*) compared to a positive stain in articular cartilage (Figure 2b*, right panel*). Toluidine Blue staining images at P4 showed different cell morphologies in fibrocartilage (a large and column-like cell) and articular cartilage (many small cells) (Figure 2c). Toluidine Blue staining images showed the “invaded” fibrocartilage drastically expanded with intense purple staining in both non-calcified and calcified areas at P33, indicating more cartilage matrix production (Figure 2d). At P60, fibrocartilage replaced essentially all existing cartilage above the 2^nd^ ossification center (Figure 2e). Together, the above data showed a close link of the invaded/expanded enthesis fibrocartilage to tendon, and that enthesis fibrocartilage had different structural features than articular cartilage.

### Drastic differences between enthesis fibrocartilage and articular cartilage at electronic microscopy (EM) levels

**Figure 3:**
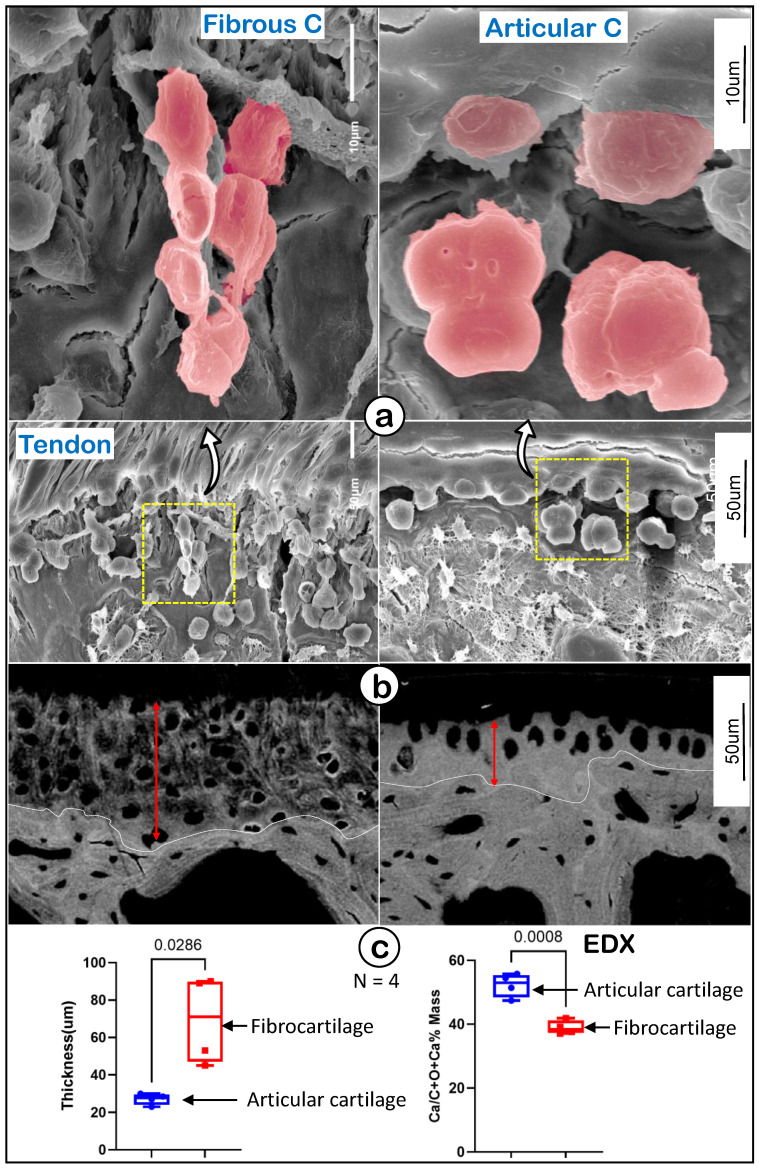
** Representative structures of mouse humerus fibrocartilage and articular cartilage at EM levels. (a)** Images of the acid-etched scanning electronic microscope (SEM) of a 12-week-old humerus head exhibited different features of fibrocartilage (left panels) and articular cartilage (right); and **(b)** the Back-scattered SEM from the same humerus head showed differences between fibrocartilage (left panels) and articular cartilage (right) in thickness, matrix structure and mineral contents; and **(c)** quantitation of thicknesses and Ca^2+^ content of fibrocartilage and articular cartilage (n = 4; p < 0.05 or 0.01). C, cartilage.

To further define the features of fibrocartilage versus articular cartilage at EM levels, we first used acid-etched scanning EM (SEM) 42 to show a spindle-like column structure in fibrocartilage compared to more round hypertrophic chondrocytes in articular cartilage (Figure 3a). Our backscattered SEM images displayed wider but poor mineral content in the fibrocartilage area (Figure 3b, *left panel*) compared to articular cartilage (Figure 3b, *right panel*). Our quantitation analyses demonstrated that those changes were statistically significant between the two groups (Figure 3c; n = 4, p < 0.05 for thickness, and p < 0.01 for mineral content as reflected by EDX measurement). These data demonstrated the unique cell morphology and mineralization in enthesis fibrocartilage, which is distinct from that of articular cartilage.

### An exclusive expression pattern of molecular markers in enthesis fibrocartilage compared to articular cartilage

**Figure 4:**
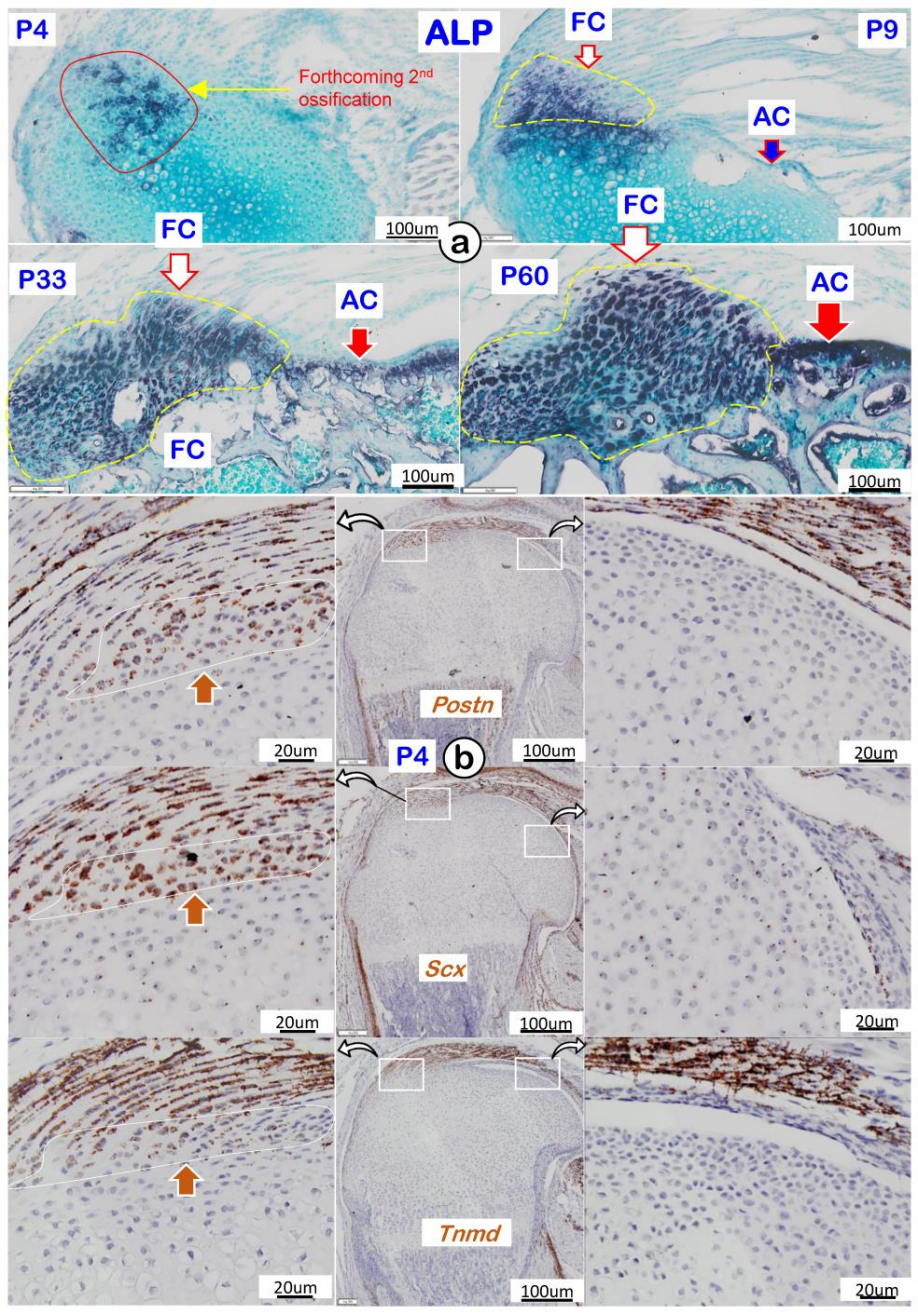
** Cellular and molecular features of mouse humerus fibrocartilage (FC) and articular cartilage (AC). (a)** Alkaline phosphatase (ALP) at different time phases with FC ahead of AC; and **(b)** RNAscope of tendon markers in the fibrocartilage cells, including *Postn* (upper panel), *Scx* (middle panel), and *Tnmd* (lower panel), which were not detected in the articular cartilage cells at postnatal day 4 (right panels).

To better characterize the features of fibrocartilage at molecular levels, we analyzed cellular alkaline phosphatase (ALP; reflecting mineralized cell activity) during four different development timelines. As shown in Figure 4a, there was essentially a lack of ALP in both fibrocartilage and articular cartilage at P4 (*upper left panel*). During development, the ALP level gradually increased and was first detected in fibrocartilage at P9, with no activity observed in articular cartilage (*upper right panel*). By P33 and P60, ALP levels further increased in both articular cartilage and fibrocartilage (*lower panels*). Of note, RNAscope assay showed strong expressions of the following tendon markers in both tendon and fibrocartilage: *Postn* (*upper left panel*), *Scx* (*left panel*), and *Tnmd* (*lower left panel*) with no signals in either the existing or articular cartilage (Figure 4b). The above data support a similar origin of tendon and fibrocartilage during postnatal development.

### Scx^Lin^ Tendon Cells Directly Contribute to Growth and Expansion of Fibrocartilage (FC) During Postnatal Development

**Figure 5:**
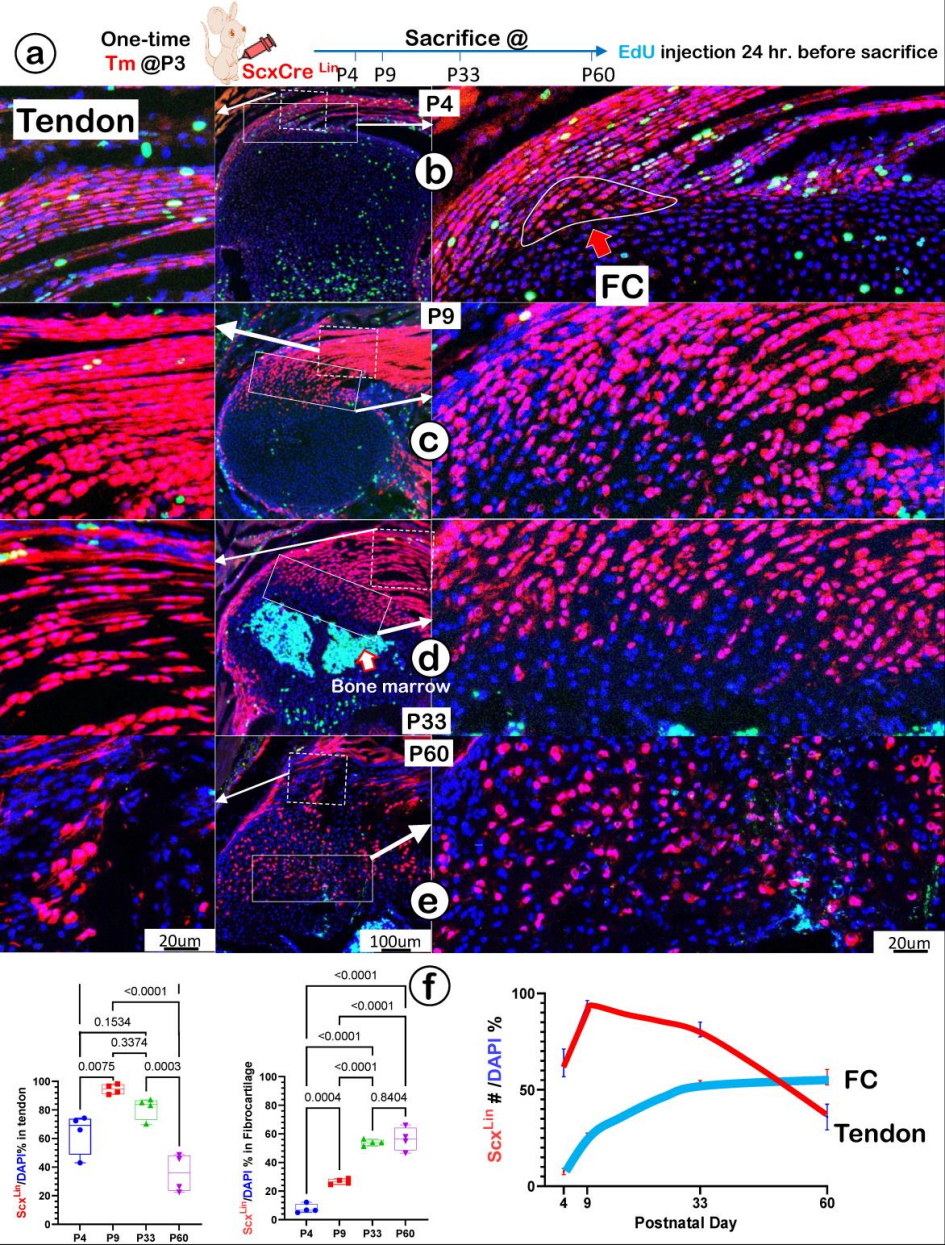
** Lineage tracing of Scx+ cells and their progeny in tendon and fibrocartilage cells. (a)** Schematic description of *ScxCreERT2*/+; *R26RtdTomato*/+ mice were induced with tamoxifen (Tm) at postnatal day (P) 3 and harvested at P4, P9, P33, and P60, separately (with one-time EdU injection 24 hr. before sacrifice). **(b-e)** The confocal images showed a biphasic change of Scx^Lin^ cells (a rapid increase and then gradual decrease) in tendon (*left panels*) and a progressive increase of tdTomato+ cells+ cells over time in a fibrocartilage (FC) area; and **(f)** The quantification of the number of tdTomato+ cells/DAPI % in tendon (*n* = 4; *left panel*) and in FC (*n* = 4; *right panel*), plus the overlap curves of the Scx+ cells % in tendon and FC.

To precisely define the FC cell origin, we generated a Scx^Lin^ cell line by crossing Scx^CreERT2^ (a line widely used for tracing the cell fate of tendon) 43 with R26R^tdTomato^. A single tamoxifen injection was given at P3, and mice were harvested at P4, P9, P33, and P60, respectively. In addition, a one-time EdU injection was performed 24 hours before the mouse sacrifices (Figure 5a). At P4, there were numerous Scx^Lin^ cells plus more EdU^+^ signals in tendon (Figure 5b, *left panel*) but few Scx^Lin^ cells in a small FC area (Figure 5b, *right panel*). At P9, there was a sharp increase in Scx^Lin^ cells in both tendon (Figure 5c, *left panel*) and the FC area (Figure 5c, *right panel*). By P33 (Figure 5d) and P60 (Figure 5e), there was a further expansion of Scx^Lin^ cells in FC (*right panels*) but a progressive reduction in tendon (*left panels*). Our quantitative data showed that the increased Scx^Lin^ FC cells (as reflected by Scx^Lin^ /DAPI %) were statistically significant between P4 and the rest of the group in tendon (except for P33) (Figure 5f, *left panel*; n = 4) and FC (Figure 5f, *middle panel*; n = 4). Scx^Lin^ cell percentage in tendon and FC displayed different trends: a biphasic change with the initial increase but a late reduction in tendon versus a steady increase in FC. Notably, there were no Scx+ bone cells, indicating that the Scx^Lin^ fibrocartilage cells do not differentiate into bone cells (Figure 5d-f).

**Figure 6:**
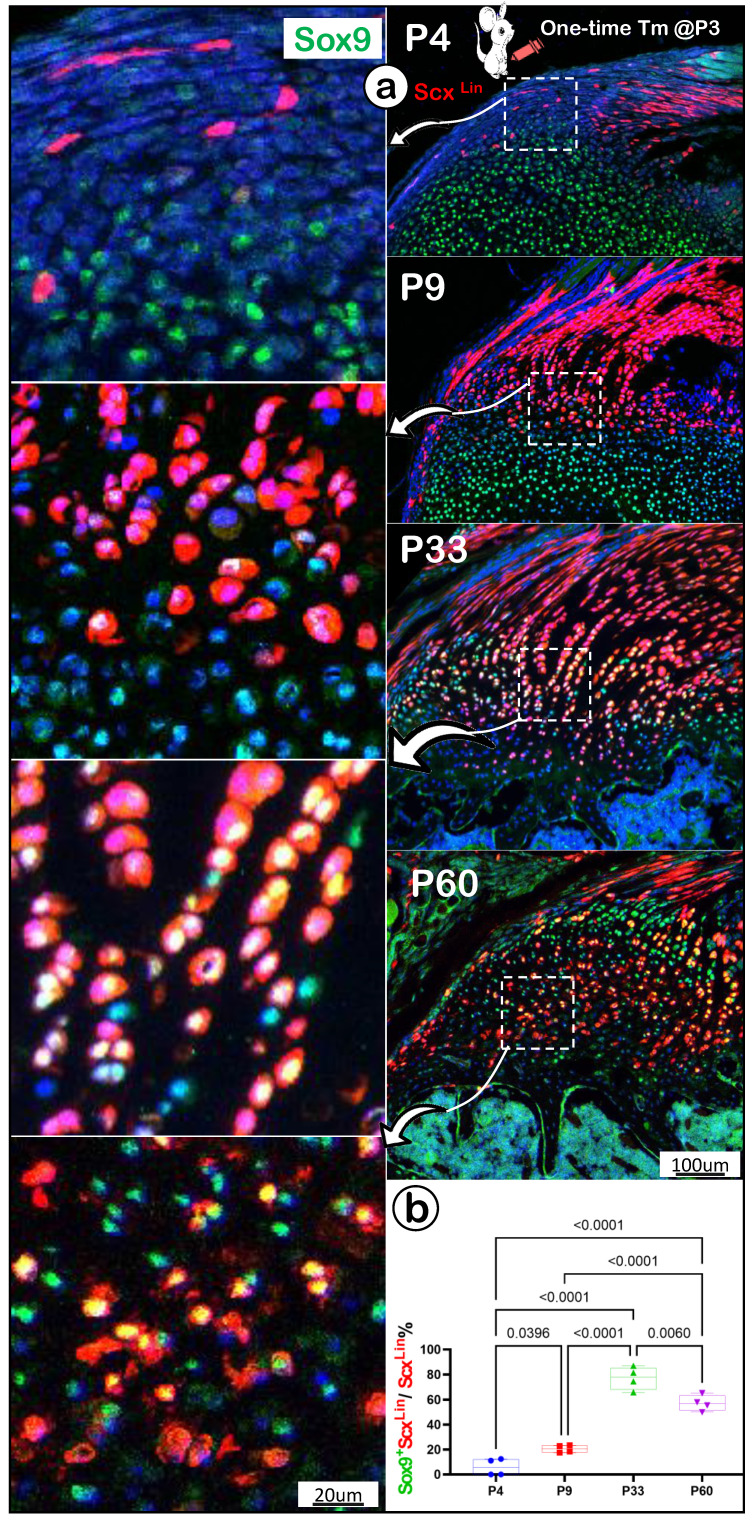
** A lack of Sox9 expression in the newly labelled Scx+ enthesis fibrocartilage cells (FC). (a)** A co-stain of Sox9 in the Scx^Lin^ mouse humerus FC cells (induced at P3 and harvested at P4, P9, P33, and P60, respectively) with no Sox9+/Scx+ FC cells at P4, but a few at P9 and sharp increases at P33 and P60; and **(b)** the quantification of the incidence of Sox9+/Scx+ FC cells among different developmental stages showed significant changes (n = 4).

To further support the FC cell origin from tendon, we co-stained Sox9 (an early cartilage marker essential for chondrocyte differentiation 44) humerus head tissue slides from P4, P9, P33 and P60. There were no Sox9+/Scx+ FC cells detected in the enthesis FC area at P4, one day after tamoxifen injection (Figure 6a, top panels), whereas Sox9+/Scx+ FC cells were observed at P9 (Figure 6a, top middle panels). By P33 and P60, many Scx+ FC cells were Sox9+ (Figure 6a, bottom panels). Quantitation data showed that these changes are statistically different among these groups, (Figure 6b), strongly supporting the cell transdifferentiation from Sox9-/Scx^Lin^ tendon cells to Sox9+/Scx^Lin^ FC cells.

Because EdU signals in tendon or FC were overall low in all groups (except at P4), we performed a PCNA immunostain assay. The test results showed a strong expression of PCNA in FC nuclei at P4 (the peak time) and gradually reduced over time with few positive FC cells at P33 and P60 (Figure S1). This information indicates that the active cell proliferation only counted for a small portion of the increased FC cell mass, which mainly took place at an early stage.

Taken together, the above data suggest that fibrocartilage cells originate from the Scx+ cells in tendon.

### Scx^Lin^ Fibrocartilage (FC) Cells progressively produce a Strong Mixture of Cartilage and Bone Extracellular Matrix (ECM) Proteins with Continuous Recruitment of Scx^Lin^ Cells during Postnatal Development

**Figure 7:**
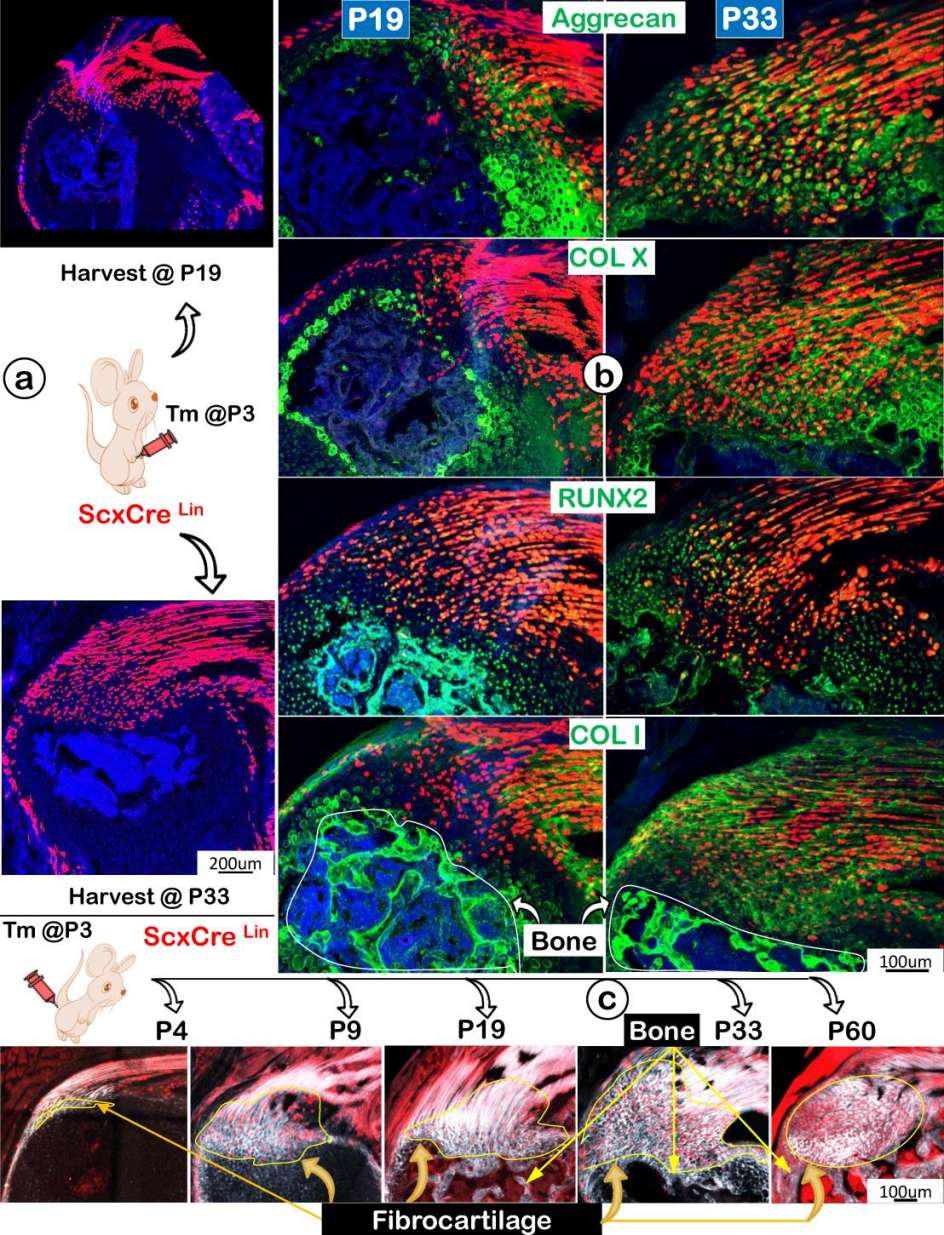
** Progressively more productions of ECM proteins and collagen fibers (as shown by SHG, second-harmonic generation) along with the increased Scx+ cells in the fibrocartilage (FC) area. (a)** One-time injection of tamoxifen (Tm) at postnatal day (P) 3 led to progressive increases in Scx^Lin^ cell numbers between P19 and P33; **(b)** The co-immunostain of different ECM markers showed a more production of cartilage matrices (aggrecan and ColX; top panels) and bone matrices (Runx 2 and Col 1; bottom panels); and **(c)** Montage of Scx^Lin^ tracing(red) and SHG(gray) images at different developmental stages: P4, strong SHG signal in tendon but a low SHG signal in the newly formed fibrocartilage (FC); P9, a continuous expansion of the Scx+ cell population and increases in SHG signal in FC with a different fibril distribution pattern from that in tendon; P19, P33 and P60, further expansion of Scx+ cells and SHG fibers in FC with no Scx+ cells in bone.

Following the Scx^Lin^ tracing studies that showed more Scx^Lin^ cells in the FC area but a decrease in tendon mass (indicating likely cell transdifferentiation from tendon to FC), we compared expression profiles of two key transcription factors (SOX9 and RUNX2) and ECMs using the same Scx^Lin^ tracing line during development. Specifically, we focused on two collection time points: P19 and P33, with a one-time injection of tamoxifen at P3 (Figure 7a). The representative immunofluorescent confocal images exhibited low expression profiles of SOX9 and RUNX2 but a high level of Aggrecan with no detectable expressions of COL X and COL I in P19 (Figure 7b, *left panels*). By P33, the expanded FC expressed high levels of these two transcription factors and ECM proteins, indicating a gradual maturation process of Scx^Lin^ cells during FC growth and expansion (Figure 7b, *right panels*). Interestingly, the co-stain of Col 1/Scx+ slide showed fibrocartilage cells do not further transdifferentiate into bone cells in both P19 and P33 (Figure 7b, lower panels). To further test this hypothesis, we studied maturation processes of type I collagen in the background of Scx^Lin^ cells from P4 to P60 taking advantage of the second-harmonic generation (SHG)-two photon technique. Our SHG imaging (Figure 7c; Figure S2) displayed different distribution patterns of type I collagen in tendon (filamentous, long threadlike structures, smooth, compact, and elongated), FC (spherical coccus, circular bundles, irregular, and round), and bone (relative low levels, irregular, relative smooth fiber distribution). Importantly, there was no red cell in bone, supporting the premise the Scx+ FC cells do not differentiate into bone cells (Figure 7c).

**Figure 8:**
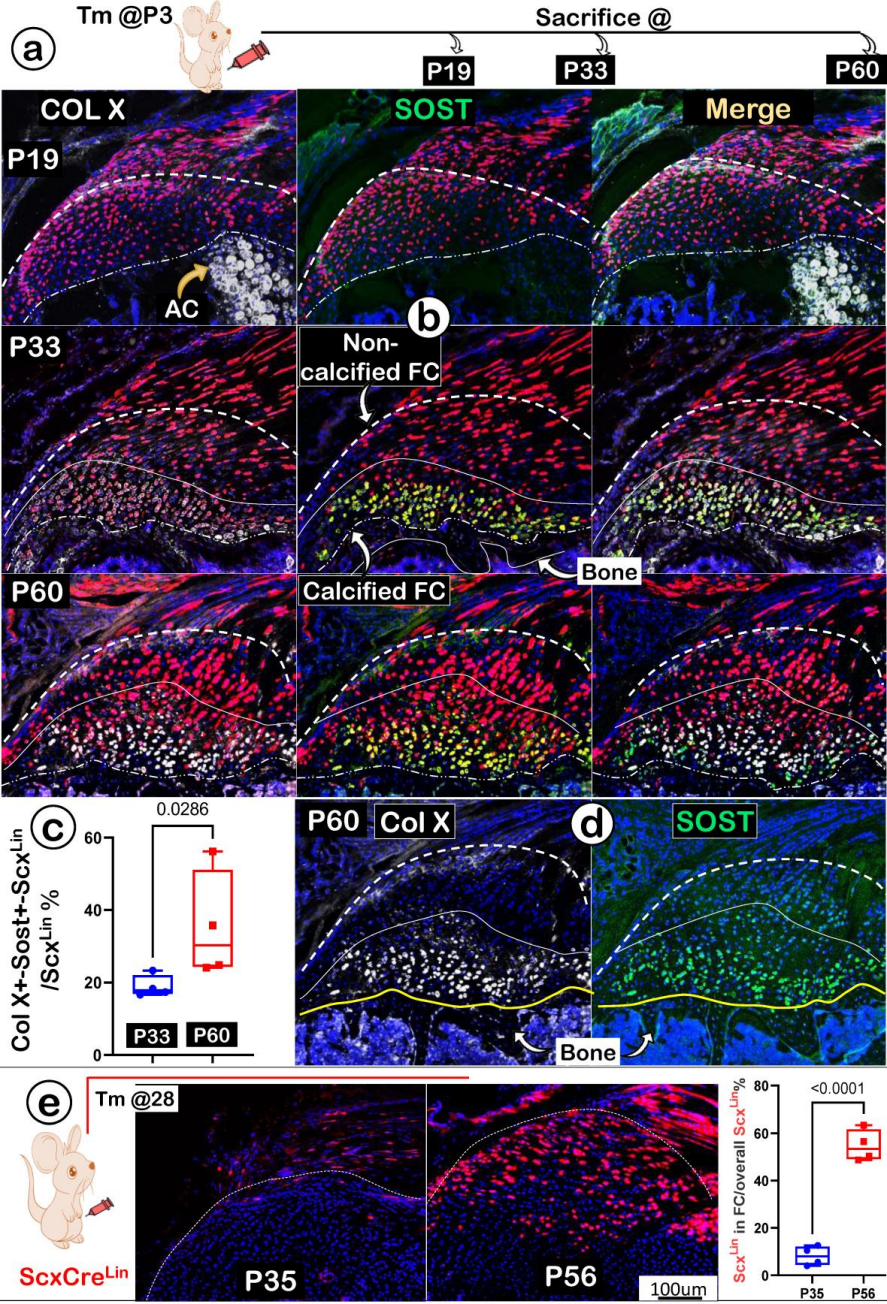
** A lengthy maturation process with continuous recruitment of Scx^Lin^ cells during fibrocartilage expansion. (a)** One-time injection of tamoxifen (Tm) at postnatal day (P) 3 followed by animal sacrificing at P19, P33 and P60, respectively; **(b)** Co-immunostain images showed a gradual increase of two ECM markers (Col X and SOST) from undetectable to the highest level in the mature calcified fibrocartilage; **(c)** Quantitation of the ratio of Col X+- Sost+-Scx^Lin^ /Scx^Lin^ % between P33 and P60 (n=4; p < 0.05); **(d)** Individual immunostain images of COL X (left panel) or SOST (right panel) for appreciation of clear pictures exhibiting strong signals in mature fibrocartilage cells at P60; and **(e)** one-time injection of tamoxifen (Tm) at P28 continuous resulted in an increase in the Scx^Lin^ positive cell numbers between P35 (+7 day) and P56 (+28 day); the quantitation of the increased Scx^Lin^ positive cell numbers between P35 (+7 day) and P56 (+28 day) (n = 4). AC; articular cartilage.

To better address the ECM maturations in FC, we studied expression levels of COL X (actively expressed in mature chondrocytes; gray color) and SOST (a potent inhibitor of Wnt signaling; highly expressed in osteocytes; green color). Again, we used one-time activated Scx^Lin^ at P3, and mice were harvested at P19, P33, and P60, respectively (Figure 8a). Our immunostain images displayed no detectable expressions of either COLX or SOST at P19 (Figure 8b;* upper panels*). By P33, noticeable co-expression of these two late markers were shown in FC cells (Figure 8b;* middle panels*). At P60, both markers were highly co-expressed in the same calcified FC cell (Figure 8b;* lower panels*). Quantitation data of co-expressions for both markers in the Scx^Lin^ fibrocartilage cells (as reflected by the ratio of Col X^+^-Sost^+^-Scx^Lin^ /Scx^Lin^) showed a significant difference between P33 and P60 (Figure 8c; n=4; p < 0.05). Notably, we observed a much higher level of SOST in fibrocartilage than that in bone cells (Figure 8d*; right panel*); this may help explain its low mineral content due to a high expression level of this local Wnt inhibitor.

Next, we asked whether there is new recruitment of Scx^Lin^ cells during FC expansion beyond the earlier labeled Scx^Lin^ tendon cells. Specifically, mice were given a one-time injection of Tamoxifen at P28 and harvested at P35 (one-week tracing) and at P56 (four-week tracing) (Figure 8e; *left panel*). The confocal Scx^Lin^ lineage tracing images showed continuous recruitment of Scx^Lin^ tendon cells with few Scx+ FC cells at P35 but much more Scx^Lin-^FC cell numbers at P56 (Figure 8e;* middle panels*). This finding was statistically significant (Figure 8e; *right panel*).

Together, the above data indicate a lengthy maturation process of fibrocartilage with a continuous recruitment of Scx^Lin^ cells during postnatal development.

### Ablation of Scx^Lin^ Cells Leads to Fibrocartilage Hypoplasia

**Figure 9:**
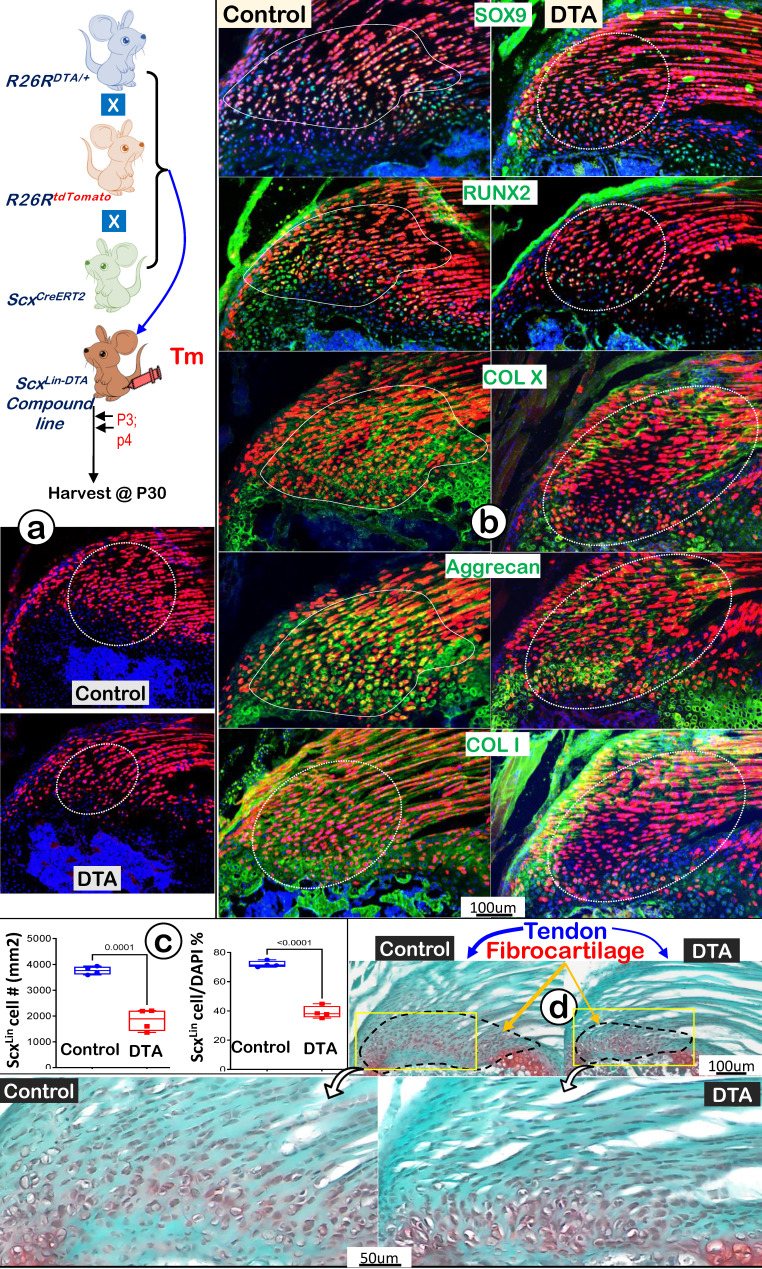
** DTA-ablation of Scx^Lin^ cells led to fibrocartilage hypoplasia. (a)** Schematic diagram of Scx^CreERT2/+^; R26R^DTA/+^; R26R^tdTomato/+^ mice with tamoxifen administration from postnatal day (P) 3-4 (once daily for two consecutive days) and harvest at P30 (upper panel). The Scx^Lin^ tracing data showed a reduction in FC cell number (lower panel); **(b)** The co-immunostain of different cartilage and bone markers showed a drastic reduction in SOX9, RUNX2, COLX, Aggrecan and COL1 in the DTA group; **(c)** The quantitation of the Scx^Lin^ positive cell # (left panel) and the ratio of the Scx^Lin^ positive cell #/DAPI cell # were significantly reduced in the DTA group (n = 4; p < 0.001); and **(d)** Representative Safranin O stain images displayed a considerable reduction in fibrocartilage mass plus cellular morphology in the DTA group.

To further support the above hypothesis that Scx^Lin^ tendon cells are the major FC cell sources for humerus head growth, we performed cell ablation assays using *Scx^CreERT2/+^*; *R26R^DTA/+^*; *R26R^tdTomato/+^* mice. Tamoxifen was administered from P3 (once a day) for 2 consecutive days, and mice were harvested at P30 (Figure 9a, *upper panel*). Our tracing data showed a decrease in both tendon and fibrocartilage cell numbers in the DTA group (Figure 9a, *lower panel*), which was statistically significant compared to the non-treated control group (Figure 9b, the DTA-treated tendon, *upper panel;* the DTA-treated FC, lower* panel;* n = 4). Co-immunostain images displayed considerable reductions in both transcription factors (SOX9 and RUNX2) and ECM proteins (COL X, aggrecan, and COL 1) in the DTA group (Figure 9c, *right panels*). Quantitative data showed significant reductions of two key markers (Sox9 for cartilage; and Runx2 for bone) in the DTA fibrocartilage (Figure S3). Representative Safranin O staining images displayed a drastic reduction in both tendon and fibrocartilage mass plus changes in cellular morphology such as cell size and distribution pattern (Figure 9d, *right panels*). Collectively, these data support the notion that Scx^Lin^ tendon cells are not only critical for tendon mass but also for fibrocartilage formation during postnatal growth.

### Constitutive Activation of β-catenin (CA-β-cat) in Scx^Lin^ Tendon Cells Accelerates Fibrocartilage Growth

**Figure 10:**
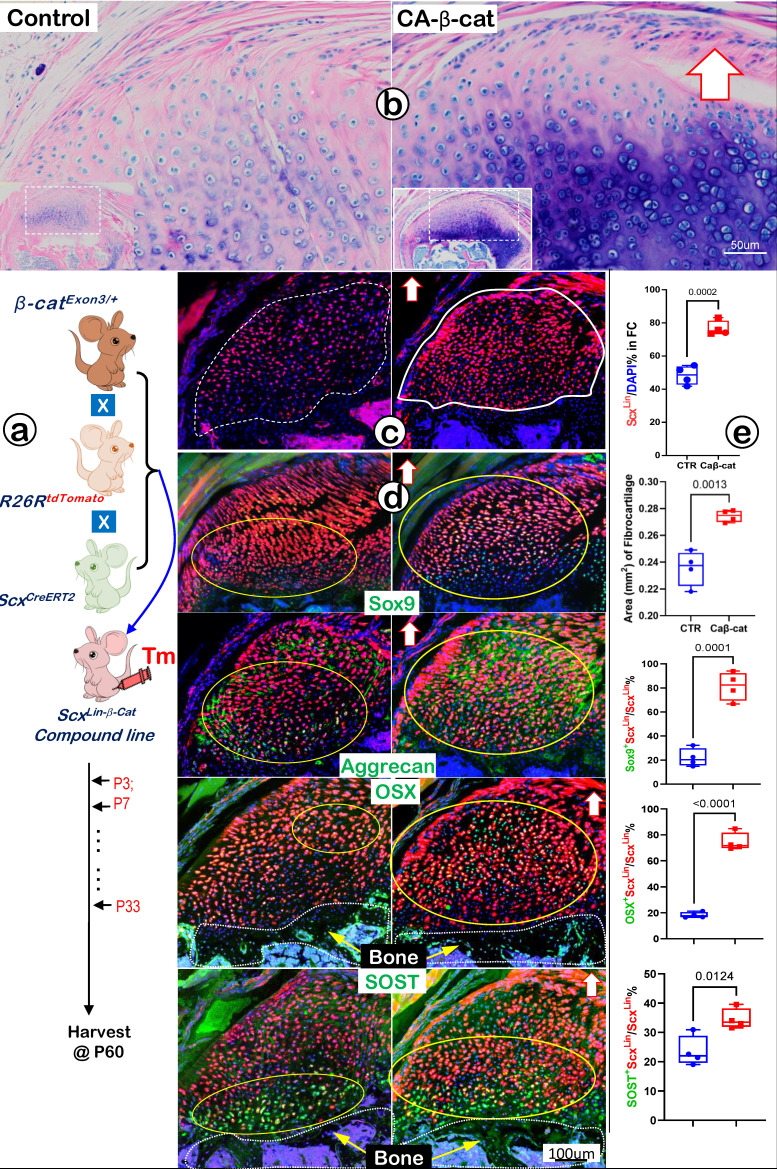
** Constitutive activation of β-catenin (CA-β-cat) in the Scx^Lin^ cells leads to expansion of fibrocartilage area and more cell numbers. (a)** Toluidine Blue shows considerably more hypertrophic chondrocytes in the CA-β-cat fibrocartilage; **(b)** Scx^Lin^ tracing images showed more red cell numbers in CA-β-cat fibrocartilage; **(c)** Co-immunofluorescent stain images revealed an increase in the expression of Sox9, Aggrecan, OSX and Sost in CA-β-cat group; and **(d)** Quantitative data shows that these increases in the cell numbers and ECM protein productions are statistically significant (n =4; p values between 0.012 and 0.0001).

Because Wnt/β-catenin signaling is critical for skeletal growth, we next created a gain-of-function model containing *Scx^CreERT2/+^*, *β*-catenin*^flox(Ex3)/+,^* and *R26R^tdTomato/+^* (CA-β-cat mouse), constitutively activated β-catenin in Scx^Lin^ tendon cells. Mice in both control and CA-β-cat groups received tamoxifen injection twice a week until P33 and were finally harvested at P60. Toluidine blue staining images displayed an excessive buildup of fibrocartilage masses with more cell numbers and strong stains in the CA-β-cat humerus head (Figure 10a, *right panel*). The tracing data showed a considerable increase in Scx^Lin^ fibrocartilage cell numbers in the CA-β-cat group (Figure 10b, *right panel*). Confocal co-immunostaining images demonstrated up-regulated expressions of both transcription factors (SOX9 and OSX) and ECM proteins (Aggrecan and SOST) in the CA-β-cat humerus head (Figure 10c, *right panels*). Our quantitative data (Figure 10d) showed a significant increase in Scx^Lin^ cell numbers and Scx^Lin^ cell mass in the CA-β-cat group. Furthermore, the quantitative data confirmed that SOX9^+^-Scx^Lin^, OSX^+^-Scx^Lin^, and SOST^+^-Scx^Lin^ fibrocartilage cell numbers significantly increased in the CA-β-cat humerus head. Together, the above data support the positive regulation of Wnt/β-catenin signaling on genes and Scx^Lin^ cell numbers during FC growth and expansion.

### 3.2 Col 1^Lin^ Mature Tendon Cells Directly Contribute to the Growth and Expansion of Fibrocartilage (FC) During Postnatal Development

**Figure 11:**
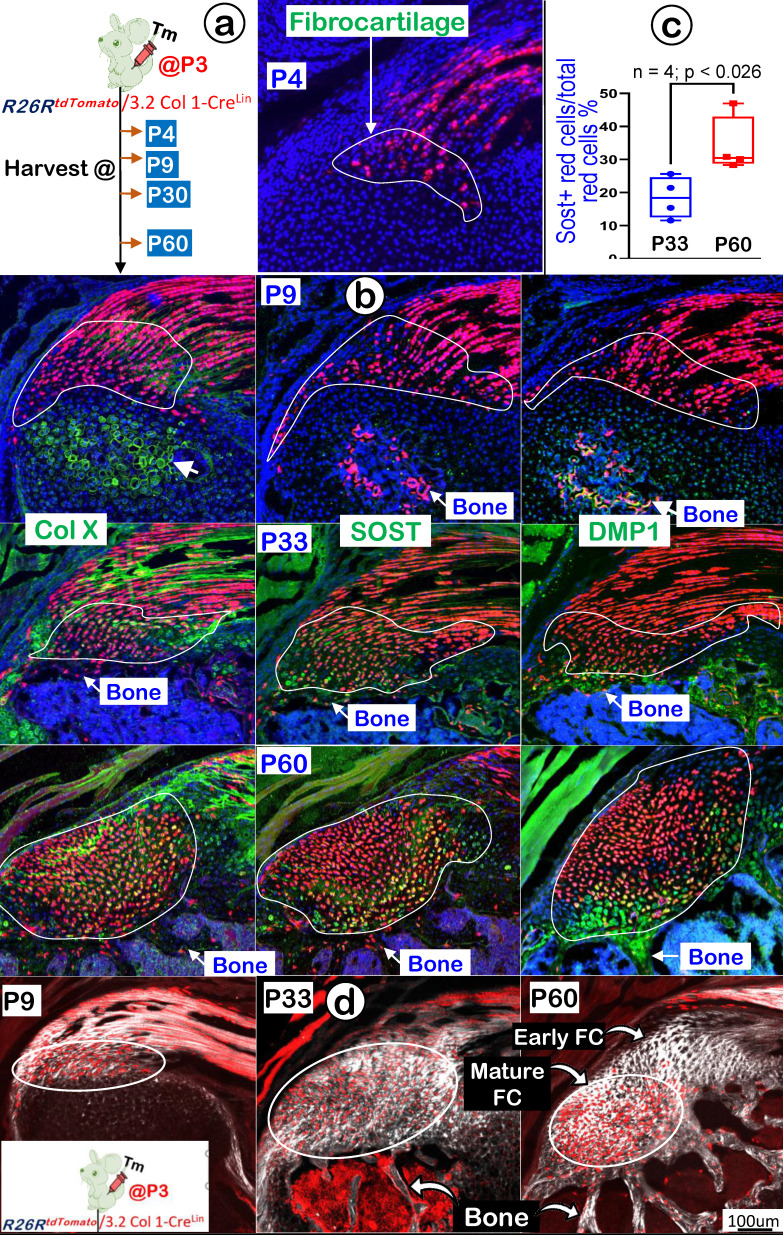
** Lineage tracing of the 3.2 Col 1+ cells and their progeny in fibrocartilage (FC) cells. (a)** Schematic description of 3.2 Col 1 ^CreERT2/+^; R26R^tdTomato/+^ mice were induced with tamoxifen (Tm) at P3 and harvested at 24 hrs., P9, P33, and P60, separately (left panel) with one-day induction signal shown on the right panel; and **(b)** The confocal images of tomato and co-immunostain of COL X, SOST and DMP1, respectively, showed a progressive increase of tdTomato+ cells over time with high levels of COL X and, SOST and DMP1 expression at P60; **(c)** The quantitation of SOST+ red cells vs total red cells in the fibrocartilage area between P33 and P60 (n = 4; p < 0.026); and **(d)** Co-images of SHG (gray color) and 3.2 Col^Lin^ signals revealed that the P3-labelled tendon cells migrate and transdifferentiate into fibrocartilage cells.

In further support of the Scx^Lin^ tracing conclusion that FC originates from tendon cells, we generated *3.2 Col 1^CreERT2/+^* ; *R26R^tdTomato/+^* mice (named 3.2 Col 1^Lin^). The mice received a one-time dose of Tamoxifen at P3 and were harvested at P9, P30, and P60, respectively (Figure 11a, *left panel*). Our one-day chasing data showed some 3.2 Col 1^Lin^ tendon cells with a considerable amount of 3.2 Col 1^Lin^ FC cells (Figure 11a, *right panel*). At P9 (6-day tracing), the co-immunostain images showed a rapid expansion of 3.2 Col 1 ^Lin^ cells in both tendon and FC as well as in the 2^nd^ ossification center (although there was no direct link between tendon-FC and bone; Figure 11b, *upper panels*). Notably, there was a low rate of Col X and a lack of DMP1 expressions in FC, but both COL X and DMP1 were strong in the 2^nd^ ossification center. By P30, there were continuous expansions of 3.2 Col 1^Lin^ cells in both tendon and FC but few 3.2 Col 1^Lin^ bone cells, indicating a separate cell origin of FC and bone (Figure 11b, *middle panels*). Importantly, we observed a noticeable SOST signal (a classic osteocyte marker 42, also a well-documented Wnt signaling inhibitor 45), in the 3.2 Col 1^Lin^ FC at this stage. By P60, there were even more 3.2 Col 1^Lin^ FC cells with strong SOST expressions (Figure 11b, *lower mid panel*). Quantitative data showed a significant difference of SOST levels between P33 and P60 (Figure 11c). On the other hand, the 3.2 Col 1^Lin^ bone cell number and SOST expression in bone remained stable between P33 and P60.

Finally, we studied maturation processes of type I collagen in the background of 3.2 Col 1^Lin^ cells from P4 to P60 combining with the SHG-two photon technique. The overlay 3.2 Col 1^Lin^/SHG imaging (Figure 11d) and the separated 3.2 Col 1^Lin^ and SHG imaging (Figure S4) revealed a continuous expansion of both 3.2 Col 1^Lin^ FC cell numbers and type one collagen mass during development. Interestingly, there were great expansion in 3.2 Col 1+ cells in the mature FC but few 3.2 Col 1+ cell numbers in the early FC and tendon, supporting the reason of the cell migration from tendon to FC (Figure 11d, right panel).

In brief, the 3.2 Col 1^Lin^ tracing data are in agreement with the Scx^Lin^ tracing studies on the origination of fibrocartilage cells from tendon and a lengthy FC ECM maturation process.

## Discussion

For many years, tendon was mainly considered a tough connective tissue that connected muscle to bone for joint movement via the fibrocartilaginous enthesis (rich in both cartilage and bone collagens such as type I and type II). Unlike articular cartilage, fibrocartilage has no perichondrium, which raises a key issue: the need to determine where fibrocartilaginous enthesis originates. In this study, we used two tracing lineage mouse lines (Scx^Lin^ and 3.2 Col I^Lin^) with comprehensive techniques to resolve this dilemma. Our work established that tendon has a direct role in forming the fibrocartilage enthesis via the cell transdifferentiation mechanism without undergoing the stem cell stage.

### Enthesis Fibrocartilage is an “Invaded" and Distinct Connective Tissue That Roots into Existing Cartilage and Firmly Anchors Tendon to Bone

Fibrocartilage (in contrast to articular cartilage) receives the least attention based on PubMed publication numbers. The former has ~1200 published articles, whereas the latter has 44,000 articles. Apparently, neglect towards fibrocartilage is not due to a lack of clinical impact, as trauma- or sports-caused damage on tendon fibrocartilage is very common, especially among young, healthy patients. A recent finding even suggested fibrocartilage as the extra-articular manifestation of rheumatoid arthritis 46. Perhaps it is the existence of confusing literature reports that make this vital but understudied tissue less attractive to readers. For example, some sources have proclaimed “no evidence of differentiation in fibrocartilage” 47 or “fibrocartilage differentiation from tendon/ligament cells at the enthesis” 48.

To better reveal the real features of fibrocartilage, we completed a side-by-side comparison between fibrocartilage and articular cartilage using both human and mouse humerus heads. By view of light microscopy, it is not surprising that dense fibers throughout the entire fibrocartilage in adult humans and mice made fibrocartilage adapt to mechanical forces; this observation included calcified and non-calcified areas (Figure 1, *left panels*). On the other hand, polarized light images showed distinct collagen fiber organization in fibrocartilage even in P4 mice with a low physical activity, reflecting a naturally occurring developmental event beyond a simple mechanical loading need (Figure 2a, *left panel*). Further histological analyses exhibited the invasion of a distinct type of tissues into the existing cartilage (i.e., round-shaped cells that were different from spindle-like tenocytes). We also observed drastic cellular changes from the initial larger and more compact cells (with low matrix contents) to smaller cells with high ECM contents at mature stages (Figures 2b-e). The EM images displayed column-like fibrocartilage cells with low mineral content but a much larger mass compared to the adjacent articular cartilage (Figure 3). Our ALP data analyses showed an increase in cellular activities from undetectable levels at early stages to a level higher than the bone underneath (Figure 4a, *left panels*). Importantly, similar mRNA expression genes including periostin, Scx, and tenomodulin in early fibrocartilage enthesis cells as well as those in tendons support the origin of fibrocartilage from tendon.

### Cell Lineage Studies Conclusively Demonstrate That Tendon Cells Form Fibrocartilage Cells in the Humerus Head

Early literature and current histological studies have already suggested the possibility that tendon cells could transdifferentiate into fibrocartilage cells. Recent cell lineage studies also indicate that Gli 1^Lin^ or Scx^Lin^ enthesis cells contribute to either patella or fibrocartilage formation 7, 10-14, 49, 50. While intriguing, these findings have not yet been considered sufficiently rigorous and conclusive to define the origin of fibrocartilage.

To precisely address the cell origin of fibrocartilage from tendon cells, we first used the following two strategies: the early-chase step (one-time activation of Scx^Lin^ at P3 followed by multiple timeline analyses; Figures. 5-7) and the later-chase step (one-time activation of Scx^Lin^ at P28 followed by 7-day and 14-day analyses, respectively; Figure 8e) during normal development. We then performed loss-or-gain-of-function studies using Scx^Lin^ tendon cells for activation of DTA (leading to a reduction in Scx+ fibrocartilage cell numbers; Figure 9) or constitutive stabilization of β-catenin (resulting in an increase in Scx^Lin^ fibrocartilage cell numbers; Figure 10). Both qualitative and quantitative data supported the idea of fibrocartilage cell origin from tendon, including more cell recruitment during later development.

Next, we demonstrated a biphasic change in Scx^Lin^ tendon cell numbers during postnatal development (an early increase followed by a gradual reduction of red labeled tendon cells); whereas Scx+ fibrocartilage cell numbers continuously expanded over time (Figure 5). Both Scx^Lin^ and 3.2 Col 1^Lin^ combined with SHG imaging data agree with these findings, further supporting the cell migration (Figures 7c and 11d). Of note, cell cycle data showed no apparent contribution to the Scx+ FC cell expansion based on the following evidences: **a**. there was a gradual reduction in cell proliferation levels within fibrocartilage cells (Figure S1); **b.** the pilot TUNEL assay data showed no apparent change among Scx+ fibrocartilage cells during early developmental stages (P4 to P33) but a sharp increase at the stage of P60 (Figure S4). Thus, we reason that fibrocartilage formation and expansion are in part a consequence of cell migration from tendon.

### The Cell Transdifferentiation Mechanism Plays a Key Role in Skeletal Development, Including the Tendon-Formed Fibrocartilage in the Humerus Head

It has been known that cell transdifferentiation from one type of mature cells to another type of mature cells occurs in gastrulation, neural crest and somite dissociation, craniofacial development, wound healing, organ fibrosis, and tumor metastasis 17, 51. Our recent publications 18, 19 agreed with studies from others 20-24 in demonstrating direct transdifferentiation from hypertrophic chondrocytes to osteoblasts/osteocytes. This pattern took place in the TMJ condyle ramus or long bone without undergoing the stem cell stage. (The changes occurred as fast as just one day!)

Although we mainly used Scx^Lin^ for tracing fibrocartilage origin in this study, we found that the 3.2 Col 1^CreERT2^ line (a mouse line highly active in mature osteoblasts 52) combining either with co-stains of different ECMs or with SHG imaging was particularly suitable for studying the direct cell transdifferentiation mechanism from tendon to fibrocartilage based on the following characteristics (Figure 11; Figure S5): 1) largely restricted in mature tenocytes; 2) quick cell transdifferentiation, which took place within 24 hours from the initial activation of the Cre event in tenocyte to red fibrocartilage cells; 3) a slow and lengthy maturation process from the newly formed 3.2 Col 1^Lin^ fibrocartilage (with no expressions of mature cartilage and bone ECMS) to terminated fibrocartilage (expressing high levels of COL X and SOST); 4) no further cell transdifferentiation from fibrocartilage to the bone beneath; and 5) an apparent cell migration from tendon to fibrocartilage with numerous 3.2 Col 1^Lin^-FC cells but few 3.2 Col 1^Lin^ tendon cells at the age of P60. Similarly, we reported that tendon cells form a subset of chondrocytes in the TMJ cartilage head, which remain as chondrocytes without further transdifferentiation into bone cells 53. In contrast, the non-tendon-derived chondrocytes did transdifferentiate into bone cells 18, 19. Currently, we do not know such a mechanism by which tendon cells transdifferentiate into different mature cells among diversified tissue environments. We speculate that the impact of various local molecules in these tissues may play a key role on the cell fate of the migrated tendon cells.

## Summary and Conclusion

**Figure 12:**
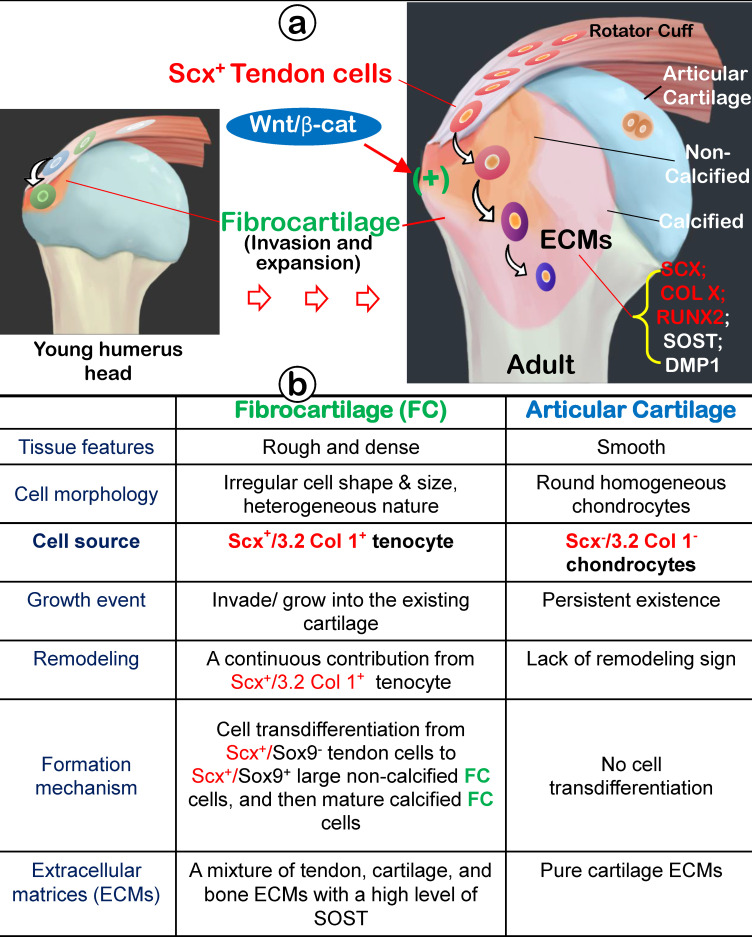
** Working hypothesis: Fibrocartilage (FC) originates from Scx+/3.2 Col 1+ tendon cells, which quickly invade and lengthily grow into the humerus epiphysis, making the tendon-bone interface an integrated continuum rather than a simple attachment.** Fibrocartilage is formed by Scx+ cells via a cell transdifferentiation mechanism, which is positively regulated by Wnt/b-catenin signaling. **(b)** Fibrocartilage, composed of non-calcified and calcified components, is distinct from articular cartilage in multiple aspects: tissue feature, cellular morphology, cell source, growth event, remodeling, formation mechanism, and extracellular matrix (ECM) proteins.

In this comprehensive study, we propose that fibrocartilage is an invaded enthesis likely originating from tendon via a quick cell transdifferentiation mechanism. The newly formed fibrocartilage roots into existing cartilage at an early stage of postnatal development and continues its lengthy growth and expansion (including the formation of non-calcified and calcified fibrocartilage plus a change in gene expression pattern). This enthesis eventually matures as a strong inside connection between tendon and bone (Figure 12) instead of acting as a simple attachment site as widely believed.

## Materials and methods

### Breeding transgenic mice

All experimental protocols followed ARRIVE (Animal Research Reporting of *In vivo* Experiments) guidelines and were assessed and approved by the Institutional Animal Care and Use Committee (IACUC) at Texas A&M College of Dentistry.

To trace Scleraxis-expressing tendon-derived cells during early postnatal fibrocartilage formation, Scx^CreERT2^ mice (provided by Dr. Ronen Schweitzer) 43 were crossed to R26R^tdTomato^ reporter mice (stock number: 009705) and named Scx^Lin^. A single intraperitoneal injection of tamoxifen (75mg/kg body weight; T5648, Sigma-Aldrich) dissolved in 90% corn oil (C8267, Sigma-Aldrich) and 10% ethanol 25 was administered at P3. Animals were sacrificed at P4, P9, P19, P33, and P60, separately, corresponding to 24 hours, 1 week, 2 weeks, 4 weeks, and 8 weeks after the tamoxifen induction. To trace the late activated Scleraxis-expressing cells during fibrocartilage formation, tamoxifen was administrated at P28, and the mice were harvested at P35 and P56, respectively, corresponding to 1 week and 4 weeks after the tamoxifen induction.

To trace Col1-expressing fibroblasts-derived cells, 3.2kb Col1^CreERT2^ mice 52 were crossed to R26R^tdTomato^ and named Col1^Lin^. Single tamoxifen was administrated at P3, and the forelimbs of mice were harvested at P4, P9, P33, and P60, corresponding to 24 hours, 1 week, 4 weeks, and 8 weeks after the tamoxifen induction.

To generate triple transgenic mice to ablate Scx^Lin^ cells conditionally, Scx^Lin^ mice were crossed with R26R^DTA/+^ mice (stock number: 009669), with the Scx^Lin^ as controls. Tamoxifen was administrated once daily for two consecutive days starting from P3, and the forelimbs were harvested at P33.

To constitutively activate β-catenin in Scx^Lin^ cells (CA-β-cat, Scx^Lin^ mice were crossed with β-catenin ^flox(Ex3)/flox(Ex3)^ mice 54. Tamoxifen was administrated twice per week, starting from P3 to P33, and then the animals were sacrificed at P60.

### Backscattered scanning electron microscopy (SEM), Acid-etched SEM and Energy Dispersive X-ray (EDX) Microanalysis

The forelimbs of 12-week-old wild type mice were fixed in 4% paraformaldehyde, dehydrated in ascending concentrations of ethanol (from 70% to 100%), and embedded in methyl-methacrylate (MMA, Buehler, Lake Bluff, IL), which were cut and polished by using 1um and 0.25um alumina alpha micropolish II solution (Buehler) as previously described 55. We used a JEOL JSM-6300 scanning electron microscope (JEOL Limited) to perform the analyses as reported previously. For acid-etched SEM, specimens were treated with 3.7% phosphoric acid for 6 s, washed twice with water, followed by two changes of 5% sodium hypochlorite for 10 min each, and rinsed again in distilled water. After that, samples were air-dried overnight and coated with gold and palladium, and were examined using a JEOL JSM-6300 SEM according to the previously described protocol 56. In addition, we acquired EDX spectrum with Ca (INCA X-sight, Oxford Instruments) detector for 60 live seconds per measured spot or area 57.

### Tissue preparation, Histology, Alkaline phosphatase activity, and Immunostaining

The forelimbs were fixed in freshly made 4% paraformaldehyde in PBS (pH7.4) for 24 ~ 48 hours and decalcified in 0.5M Ethylenediaminetetraacetic acid (EDTA) at 4 °C. Samples for histological staining and immunohistochemistry were embedded in paraffin, sectioned at 5 um thickness, and stained with Safranin O (proteoglycans), Toluidine blue, Picrosirius red 55, or Masson's trichrome stain 58. Samples for cell lineage tracing were dehydrated with 30% sucrose/PBS and embedded in an OCT compound (Sakura Tissue-Tek), cryosectioned to a thickness of 10um as previously described 59. Alkaline phosphatase (ALP) enzyme activity was measured in frozen tissue slides using an ALP Assay Kit (Roche, Indianapolis, IN, USA). The frozen sections were also used for immunofluorescent staining, which was proceeded as previously described 60 with the following antibodies: rabbit anti-Collagen I antibody (ab21286, Abcam, 1:100), rabbit anti-Aggrecan (AB1031, Millipore, 1:100), rabbit anti-Collagen X antibody (ab59632, Abcam, 1:100), rabbit anti-DMP1 antibody (kindly provided by Dr. Chunlin Qin at Texas A&M University, 1:400), rabbit anti-Sox9 antibody (AB5535, Abcam, 1:100), rabbit anti-OSX antibody (ab209484, Abcam, 1:200), rabbit anti-Runx2 antibody (ab23981, Abcam, 1:100), goat anti-SOST antibody (AF1589, R&D, 1:50), goat anti-Periostin antibody (AF2955, R&D, 1:100). The immunofluorescent signals were detected with the corresponding Alexa second antibody (Thermofisher; 1:200) at room temperature for 2 hours.

### Cell proliferation

For cell proliferation, 5-ethynyl-2'-deoxyuridine (EdU) (Invitrogen A10044) dissolved in PBS was administered to mice 24 hours before sacrifice. Click-iT Imaging Kit with Alexor Flour 647-dye (C10340) was used to detect EdU in frozen sections 61, 62. As a supplement, rabbit anti-PCNA antibody (24036-1-AP, Proteintech, 1:1000) was used to detect proliferative cells in enthesis fibrocartilage as previously described 63, 64.

### *In situ* Cell Apoptosis (TUNEL Assay)

To assess apoptotic cell death, *In situ* Cell Death Detection Kit, Fluorescein (TUNEL assay) was used with cryosections of mice specimens according to manufacturer's instructions (Roche, 11684795910). Images were taken by Sp8 confocal microscope (Leica Biosystems) at 488 (green) um-wavelength 65.

### RNAscope *In situ* Analysis

RNAscope was performed using the RNAscope 2.5 HD Brown Reagent Kit (322300, Advanced Cell Diagnostics, Neward CA, USA) on 5-um FFPE tissue sections prepared from the postnatal 5-day-old mice forelimbs according to the manufacturer's instructions. Slides were baked overnight at 60℃ before use. After deparaffinization and dehydration, the tissues were air-dried and treated with a peroxidase blocker before boiling at 90-95℃ in target retrieval reagents for 15 min. Protease was then applied for 30 min at 40℃. Target probes Tnmd, Postn, and Scx (430531, 418581, 439981, Advanced Cell Diagnostics) were hybridized for 2h at 40 °C, followed by a series of signal amplification and washing steps. All hybridizations at 40 °C were performed in a HybEZ Hybridization System. RNA staining signal was identified by DAB as brown chromogenic dots. Following the RNAscope assay, samples were counterstained for 2 min with hematoxylin. Each specimen was quality controlled for RNA integrity with a probe specific to the housekeeping gene cyclophilin B (PPIB); only specimens with an average of >4 dots per were included for analysis. Negative control background staining was evaluated using a probe specific to the bacterial dapB gene; only samples with an average of <1 dot per 10 cells were included for analysis. Bright-field images were acquired by Olympus CKX41 inverted microscope using an x20 objective 66.

### Quantitation and Statistical Analysis

All sections (MMA embedding, paraffin embedding, and OCT embedding) were made in the same direction (sagittal plane, from lateral to lateral) and selected the representative sections of enthesis among serial sections.

To outline the gradient structure of enthesis (inserted tendon, fibrocartilage and bone), tissue sections were imaged by second harmonic generation (SHG, which displays type I collagen enriched tissue) with 880 nm pulsed near infrared (NIR) excitation using the non-descanned detector under a Leica Stalaris Multiphoton Laser Scanning Confocal Microscope^67^, ^68^. To quantify the number of tdTomato+ cells, or Sox9^+^, OSX^+^, Col X^+^, Sost^+^ (IHC staining) or apoptotic cells (TUNEL assay) and the overall number of cells (counterstained with 4',6-diamidino-2-phenylindole [DAPI]) in tendon or fibrocartilage, SP8 Leica confocal microscope was used to photograph the tissue sections. All images were obtained at wavelengths ranging from 405 (blue)-488 (green)-561 (red)-633 (gray) um. Multiple stacked photos were taken at 200Hz and dimensions of 1024x1024 53. For IHC staining, the red color reflected by the tdTomato signal indicated the descendant cells with Cre-activity; green or gray color represented the corresponding immunofluorescent staining; blue color was DAPI staining. For the TUNEL assay, the green color meant apoptotic cell death. The number of tdTomato^+^, Sox9^+^, OSX^+^, Col X^+^, Sost^+^, apoptotic - and DAPI^+^ cells in a specified region of interest (tendon or FC, the fibrocartilage) was counted. The area of the specified region (FC) was measured.

For quantitating the PCNA^+^ cells (DAB, brown dots) versus overall fibrocartilage cells (counterstained by Methyl Green), bright field photos were taken by Olympus CKX41 inverted microscope using an x20 objective.

Histomorphometry measurements were performed using ImageJ software (National Institutes of Health). Four animals per group were used (n=4), with at least 4 comparable sections from each mouse. The results are expressed as boxplots (Min to Max, all points are shown). GraphPad Prism 9 software (GraphPad Software, Inc., La Jolla, CA, USA) was used for statistical analysis. Comparison between two groups was assessed by unpaired Student's t-tests. Comparisons among three or more groups were evaluated by one-way ANOVA with Bonferroni post hoc test. Unpaired nonparametric tests (Mann-Whitney) were used when the data were not normal. All P values are shown. A value of P < 0.05 was considered statistically significant.

## Highlights

- Fibrocartilage is distinct from the adjacent articular cartilage at cellular and ECMs, including a high expression of SOST (a classic osteocyte marker and a potent inhibitor of Wnt signaling);
- Scx^Lin^ tendon cells directly contribute to fibrocartilaginous enthesis formation via a quick cell transdifferentiation mechanism with a lengthy ECM maturation process;
- Tendon cells root into the humeral bone head through fibrocartilaginous enthesis for a firm connection, instead of a simple attachment manner as commonly believed;
- Demonstration of a new application of the 3.2 Col 1^CreERT2^ (a widely used bone tracing line) in studies of tendon cell fate;
- SHG (second-harmonic generation) imaging offers a valuable tool for distinctive distribution patterns of type I collagen in the tendon, fibrocartilage, and bone.

## Supplementary Material

Supplementary figures.
