# Supplementary material for: Tendon Cells Root Into (Instead of Attach to) Humeral Bone Head via Fibrocartilage-Enthesis
Source: Int J Biol Sci. 2023 Jan 1;19(1):183–203. doi: 10.7150/ijbs.79007 (PMC9760439; doi:10.7150/ijbs.79007)
Supplement: Supplementary file 1 — Supplementary figures. [file ijbsv19p0183s1.pdf]

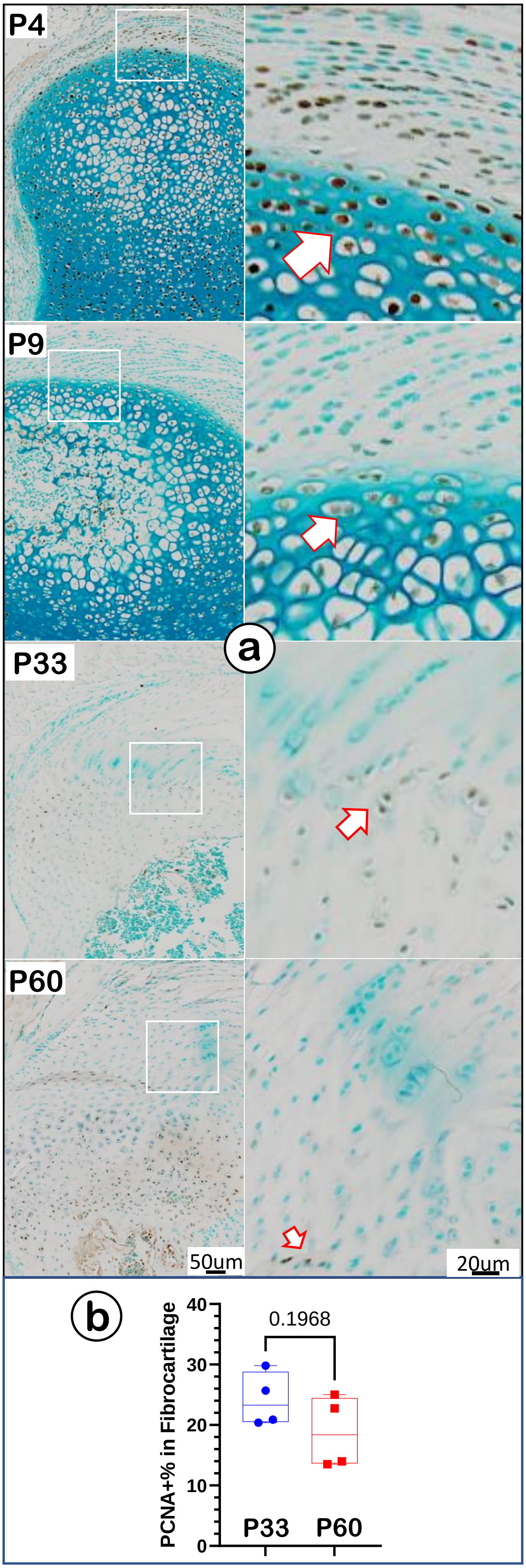

Figure S1. (**a**) Representative immunostain images of PCNA from samples collected at P4, P9, P33, and P60. (**b**) The quantitative data showed a progressive decrease in PCNA levels in fibrocartilage during humerus head development.

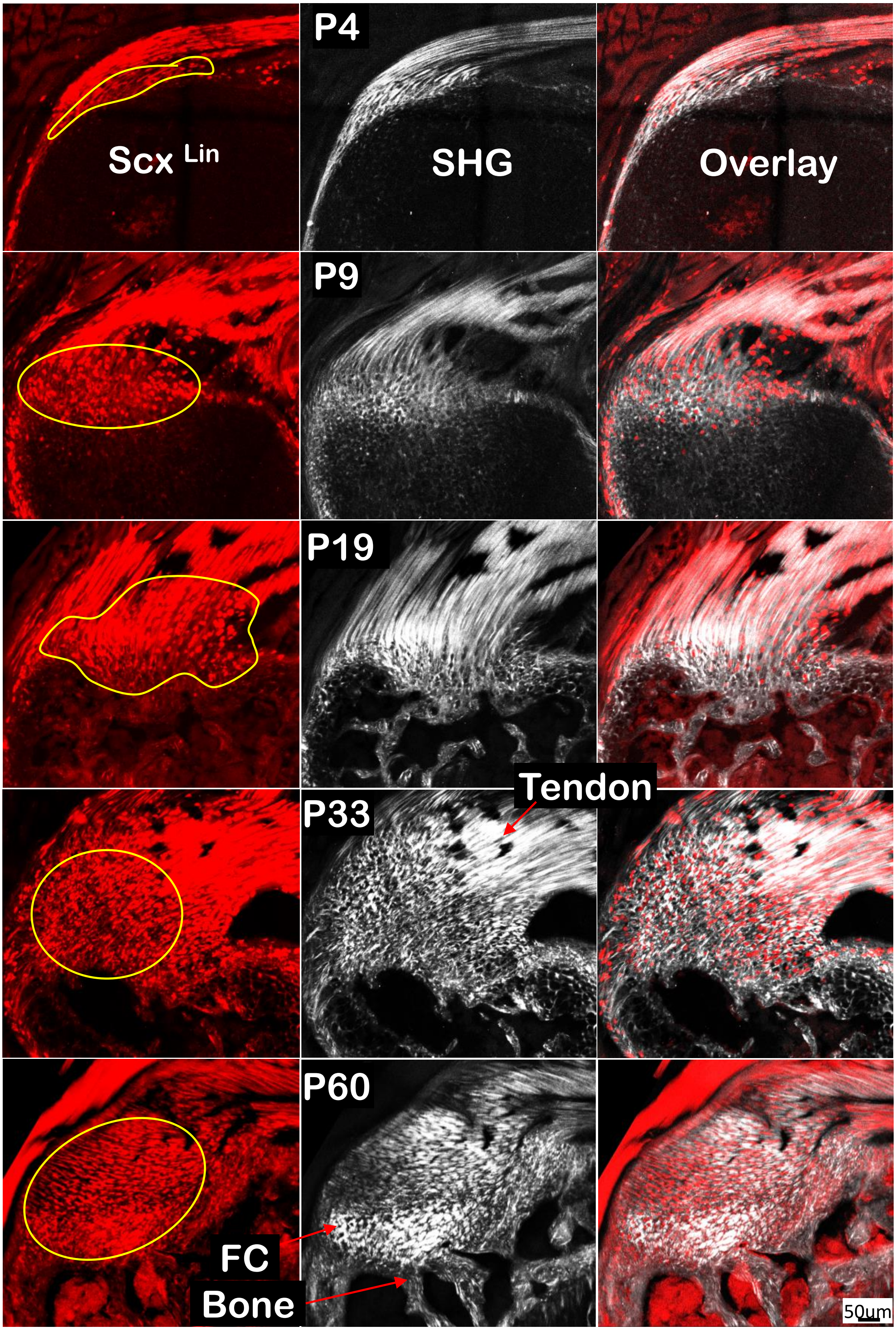

Figure S2. The SHG images provided a valuable tool to distinct unique distributions of type I collagen in tendon, fibrocartilage (FC), and bone. The combination of SHG and Scx^{Lin} tracing images further support the FC cell origin from tendon without further differentiation into bone cells.

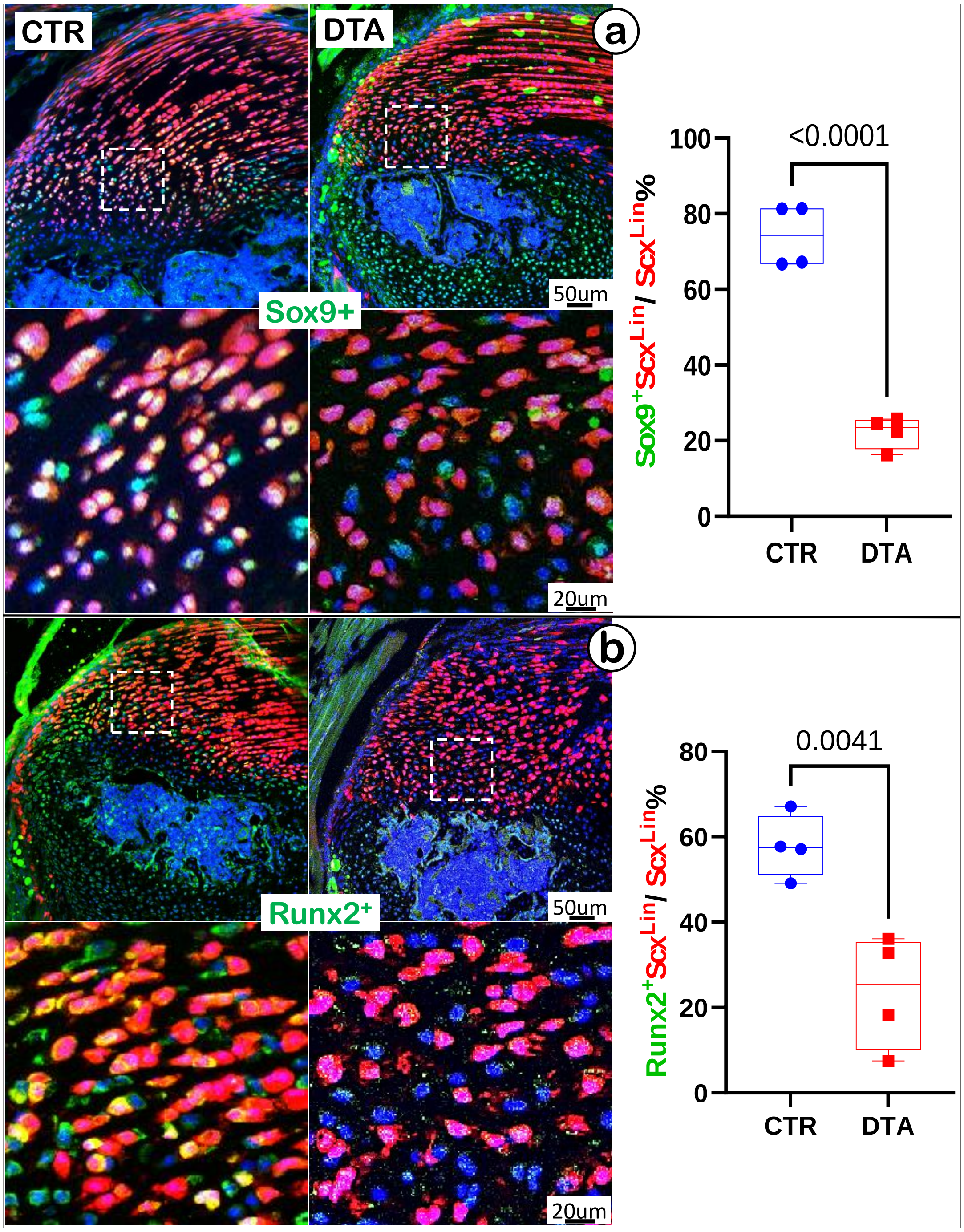

Figure S3. DTA-ablation of Scx^{Lin} cells led to significant decrease in expression levels of Sox9 and Runx2 in fibrocartilage cells with tamoxifen administration from postnatal day(P) 3&P4 (once daily for 2 consecutive days) and harvest at P30. (**a**) The co-immunostain of Sox9 showed a sharp decrease of Sox9 expression in the Scx^{Lin} fibrocartilage cells in the DTA-treated group, which is significantly different from the control (n = 4; $p < 0.001$); and (**b**) The co-immunostain of Runx2 displayed a drastic reduction of Runx2 expression in the Scx^{Lin} fibrocartilage cells in the DTA-treated group, which was significantly different from the control (n = 4; $p < 0.01$).

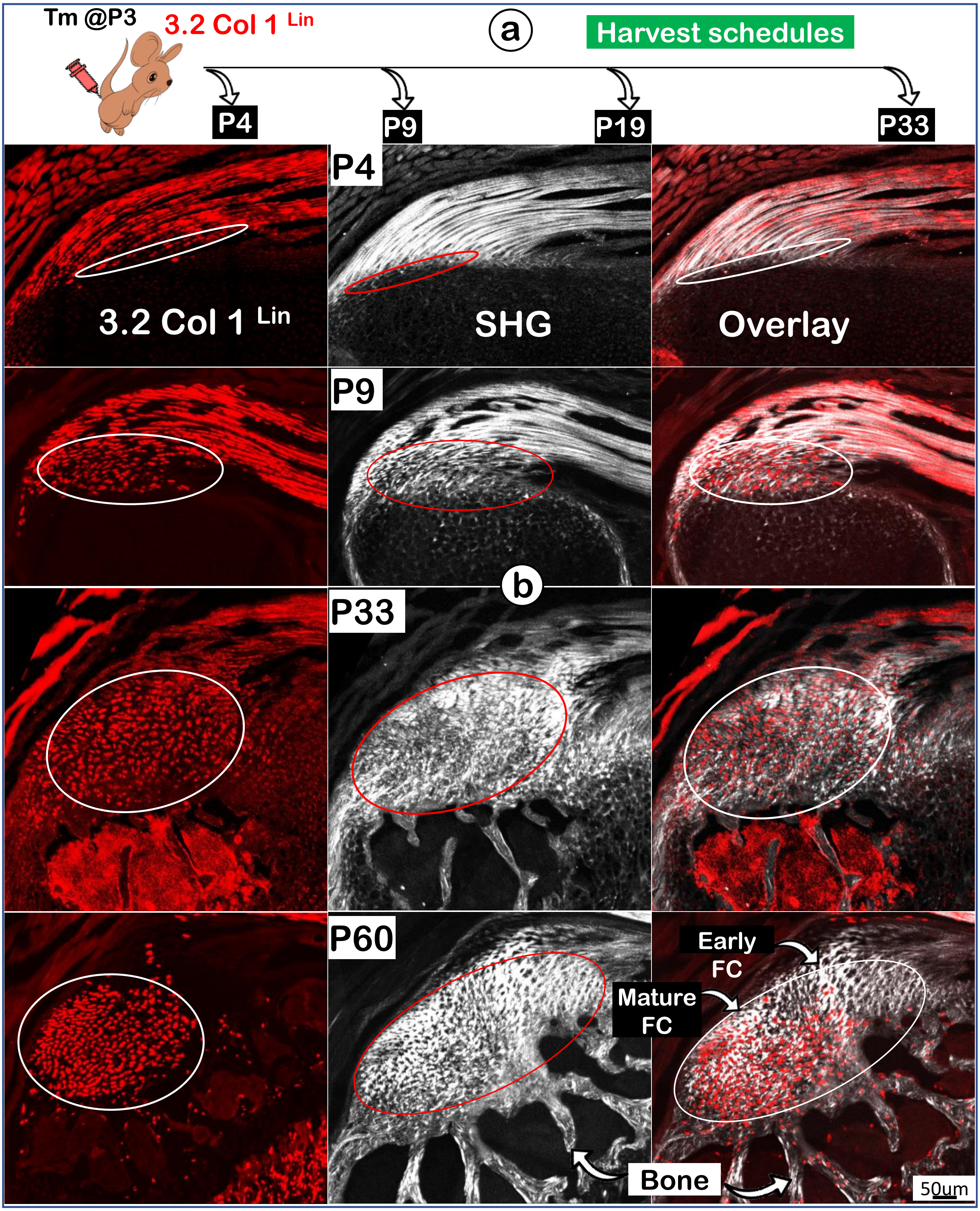

Figure S4. The SHG images in the background of the 3.2 Col 1^{Lin} displayed a close association of the 3.2 Col 1^{+}-FC cell numbers and the expansion of type I collagen mass during postnatal development. (**a**) 3.2 Col 1^{Lin} mice were induced at P3 and harvested at P4, P9, P33 and P60, respectively; and (**b**) There was a continuous increase in the 3.2 Col 1^{+}-FC cell numbers and the expansion of type I collagen mass during postnatal development but a gradual reduction of 3.2 Col 1^{+}-tendon cells during development at the stages of P33 and P60 with no 3.2 Col 1^{+} cells in the FC adjacent to tendon at P60, indicating a cell migration.

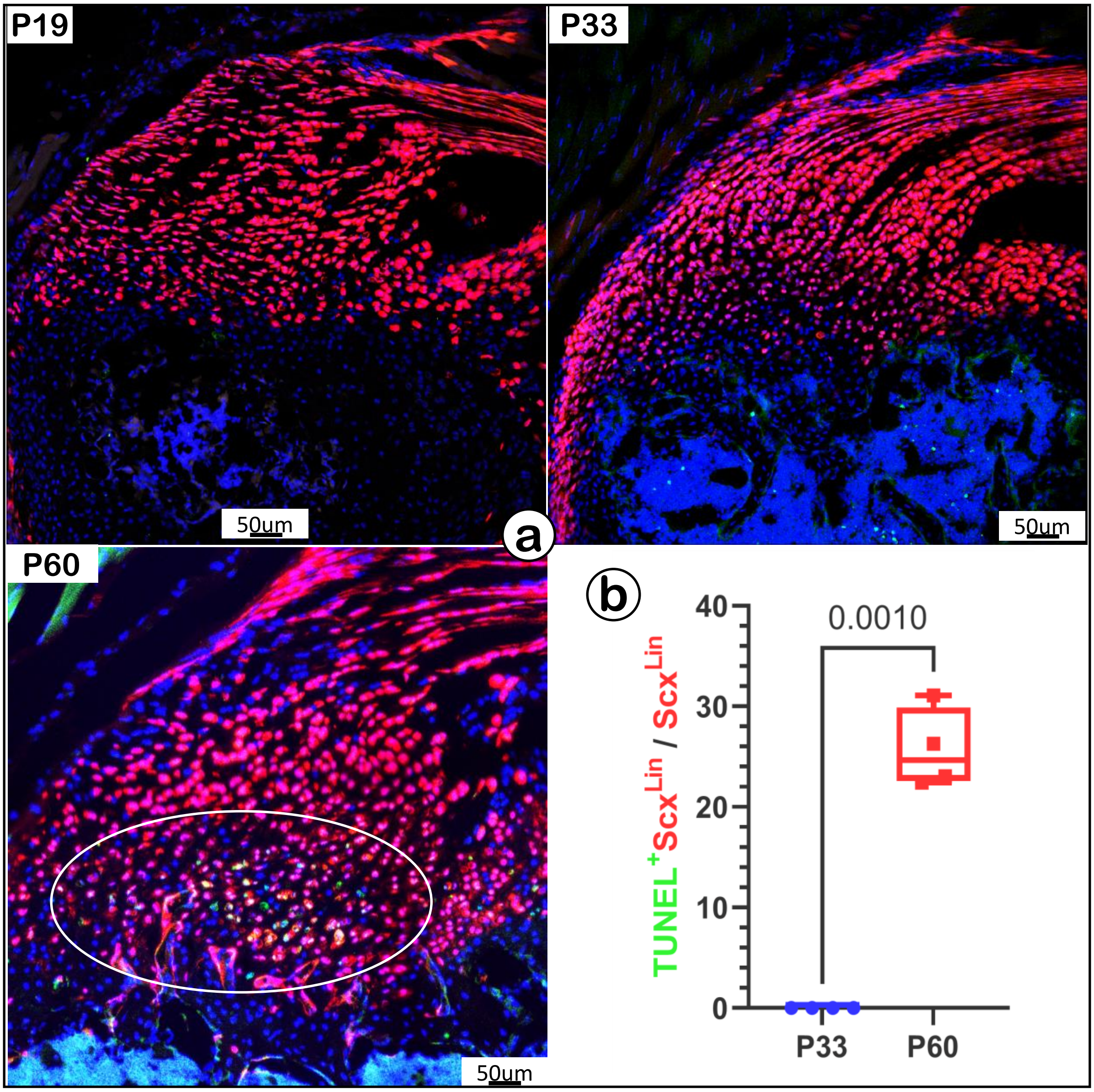

Figure S5. (**a**) Representative TUNEL assay data showed no TUNEL signals among Scx+ fibrocartilage cells during early developmental stages (P19 and P33) but a sharp increase in the TUNEL+/Scx+ fibrocartilage cells at the stage of P60; and (**b**) there was a significant difference of TUNEL levels between P33 and P60 ($p < 0.001$; $n = 4$).
